# Risk of cardiovascular events and mortality associated with LDL and non-HDL cholesterol levels after statin initiation for primary prevention: A multiregional cohort study

**DOI:** 10.1371/journal.pmed.1005012

**Published:** 2026-11-03

**Authors:** Joseph Edgar Blais, Ralph Kwame Akyea, William Harrison, Amy Hai Yan Chan, Wenfang Zhong, Stephen Franklin Weng, Kebede Abera Beyene, Wallis C. Y. Lau, Kenneth K. C. Man, Nadeem Qureshi, Esther Wai Yin Chan, Ian Chi Kei Wong, Jeff Harrison

**Affiliations:** 1 Centre for Safe Medication Practice and Research, Department of Pharmacology and Pharmacy, Li Ka Shing Faculty of Medicine, The University of Hong Kong, Pokfulam, Hong Kong SAR, China; 2 Precision Healthcare and Drug Safety (PRISM) Research Group, Centre for Academic Primary Care, School of Medicine, University of Nottingham, Nottingham, United Kingdom; 3 Medicines Intelligence Research Group, School of Pharmacy, Faculty of Medical and Health Sciences, The University of Auckland, Auckland, New Zealand; 4 GSK R&D Global Headquarters, London, United Kingdom; 5 Research Department of Practice and Policy, School of Pharmacy, University College London, London, United Kingdom; 6 Centre for Medicines Optimisation Research and Education, University College London Hospitals NHS Foundation Trust, London, United Kingdom; 7 Accident and Emergency Department, Queen Mary Hospital, Pok Fu Lam, Hong Kong SAR, China; 8 The University of Hong Kong Shenzhen Institute of Research and Innovation, Shenzhen, China; 9 Macau University of Science and Technology, Macau SAR, China; 10 Aston Centre of Excellence in Pharmacotherapy Optimisation and Pharmacoepidemiology Research, Aston Pharmacy School, Aston University, Birmingham, United Kingdom; Peking University, CHINA

## Abstract

**Background:**

The cardiovascular and mortality benefits of achieving guideline-recommended concentrations of low-density lipoprotein (LDL) and non-high-density lipoprotein (non-HDL) cholesterol with statins in primary prevention remain uncertain.

**Methods and findings:**

In this new-user cohort study, we used electronic healthcare databases from Hong Kong (HK), New Zealand (NZ) and the United Kingdom (UK) to identify 185,237 patients initiating statins between 1 January 2007 and 31 December 2012 (with follow-up to 31 December 2017) without atherosclerotic cardiovascular disease, heart failure, or chronic kidney disease. Associations between LDL and non-HDL cholesterol measured within 2 years post-initiation with the outcomes of major adverse cardiovascular events (MACE) or all-cause mortality were assessed in landmark analyses using multivariable Cox regression adjusted for age, sex, hypertension, atrial fibrillation, diabetes mellitus, dyslipidaemia, obesity, smoking, alcohol misuse, rheumatoid arthritis, systemic lupus erythematosus and connective tissue disorders, severe mental illness, antihypertensive drugs, antidiabetic drugs, antiplatelet drugs, and anticoagulant drugs. Estimates were pooled using random-effects meta-analysis. At 2 years, LDL cholesterol was <1.4 mmol/L in 7,084 (3.8%), 1.4–1.7 mmol/L in 21,893 (11.8%), 1.8–2.5 mmol/L in 81,941 (44.2%) and ≥2.6 mmol/L in 74,294 (40.1%) patients. Mean duration of follow-up for the primary outcome of MACE was 6.5 years in HK, 5.6 years in NZ and 5.4 years in UK. versus an LDL cholesterol ≥2.6 mmol/L, adjusted hazard ratios (HR) for MACE were 0.80 (95% CI [0.77,0.83]) for 1.8–2.5 mmol/L, 0.79 (0.66,0.94) for 1.4–1.7 mmol/L and 0.82 (0.60,1.12) for <1.4 mmol/L. Non-HDL cholesterol showed similar estimates for MACE (e.g., HR 0.75, [0.72,0.79] for 2.6–3.3 mmol/L versus ≥3.4 mmol/L). In continuous analysis, the association with MACE was approximately log-linear across achieved LDL and non-HDL cholesterol in HK and the UK, whereas in NZ estimates were less consistent and imprecise at the lowest concentrations. After adjustment for confounding factors, very low LDL (HR 1.06, [0.96,1.16]) and non-HDL cholesterol (HR 0.98, [0.84,1.14]) were not associated with all-cause mortality. Limitations include heterogeneity across cohorts, which may reflect confounding by indication where treatment intensity was guided by cardiovascular risk, and residual confounding from comorbidities, lifestyle interventions and disease severity. Estimates are also conditional on patients having initiated statins, survived to the landmark, and undergone follow-up lipid testing.

**Conclusions:**

Lower achieved LDL and non-HDL cholesterol after statin initiation was associated with a reduction in MACE, with limited evidence of an increase in all-cause mortality at very low concentrations after adjustment for confounding. Additional reductions in cardiovascular events could be obtained by achieving guideline-recommended non-HDL cholesterol levels of at least <3.4 mmol/L after statin initiation for primary prevention.

## Author summary

**Why was this study done?:**

- While lower levels of low-density lipoprotein (LDL) and non-high-density lipoprotein (non-HDL) cholesterol are generally linked to fewer cardiovascular events such as heart attacks and strokes, some prior studies raised concerns that very low cholesterol levels might increase the risk of death.
- Previous research had significant gaps: it rarely focused on people at lower cardiovascular risk using statins to prevent heart disease for the first time, known as “primary prevention”, and it often did not properly account for when cholesterol levels were measured after starting a statin.
- In people who start statins for primary prevention, we needed large-scale real-world evidence on the effects of achieving very low cholesterol levels on cardiovascular events and death.

**What did the researchers do and find?:**

- We analyzed electronic health data from three large cohorts, representing a diverse population of over 185,000 adults who started taking statins to prevent their first cardiovascular event.
- We found that lower levels of LDL and non-HDL cholesterol two years after starting a statin were consistently linked to a lower risk of major adverse cardiovascular events.
- Very low cholesterol levels (LDL cholesterol <1.4 mmol/L or non-HDL cholesterol <2.2 mmol/L) were not clearly associated with a higher risk of death from any cause after accounting for other patient factors, although estimates were imprecise and differed between cohorts.

**What do these findings mean?:**

- Lower concentrations of LDL and non-HDL cholesterol after statin initiation for primary prevention were associated with fewer cardiovascular events. After adjustment, there was no clear evidence of increased mortality at very low concentrations.
- These findings support achieving guideline-recommended targets for non-HDL cholesterol <3.4 mmol/L or LDL cholesterol <2.6 mmol/L within 2 years of starting a statin.
- While this study is applicable to many patients taking statins for primary prevention, limitations include heterogeneity across cohorts and potential residual confounding by disease severity and by indication for more intensive lowering of LDL and non-HDL cholesterol.

## Abstract 3

Joseph Edgar Blais and colleagues analysed data from more than 185,000 statin users across Hong Kong, New Zealand, and the UK, finding that lower achieved LDL and non-HDL cholesterol levels after statin initiation were associated with lower rates of major cardiovascular events in primary prevention.

## Introduction

Clinical practice guidelines for dyslipidaemia recommend achieving absolute blood concentrations of low-density lipoprotein (LDL) and non-high-density lipoprotein (non-HDL) cholesterol as the primary goal of therapy after initiating statins. High levels of atherogenic cholesterol (defined hereafter as LDL and non-HDL cholesterol) are a leading modifiable cause of atherosclerotic cardiovascular disease (ASCVD) [1–3]. Treatment goals for atherogenic cholesterol have progressively moved lower in patients with established atherosclerotic cardiovascular disease (ASCVD), yet achieving very low levels in primary prevention remains highly controversial [4–6].

The relationship between on-statin treatment LDL and non-HDL cholesterol and clinical outcomes from both seminal meta-analyses of statin randomized controlled trials (RCTs) and epidemiological studies have several important limitations. In two meta-analyses of individual participant data from statin RCTs, Boekholdt and colleagues demonstrated that lower LDL and non-HDL cholesterol after statin treatment were associated with reductions in cardiovascular events [7,8]. However, most patients enrolled in these trials had established ASCVD, making the interpretation and applicability of this evidence to primary prevention unclear. Furthermore, observational studies of statin users have suggested that very low LDL and non-HDL cholesterol is associated with an increase in all-cause mortality [9–11]. These reported associations for all-cause mortality are prone to bias because the inclusion of prevalent statin users does not allow for controlling confounders at the time of statin initiation or clearly defining the timing of LDL or non-HDL cholesterol measurement after statin initiation.

Whether the risk of cardiovascular events can be further reduced through achieving guideline targets for atherogenic cholesterol in primary prevention is critically important to understand, because LDL and non-HDL cholesterol can now be effectively reduced by combining statins with non-statin medications such as ezetimibe, bempedoic acid, and PCSK9 inhibitors [12]. Uncertainties also exist about whether there is a threshold effect where most of the benefit is obtained for major adverse cardiovascular events (MACE) and all-cause mortality at specific LDL and non-HDL cholesterol concentrations while on statin treatment. Given the uncertainty and imprecision of estimates from single cohort studies and the variability in prescribed statin drugs and doses across health systems, large-scale evidence from geographically diverse populations is required. Therefore, we used real-world data from Hong Kong (HK), New Zealand (NZ) and the United Kingdom (UK) to assess the association between achieved concentrations of atherogenic cholesterol and the occurrence of MACE and all-cause mortality among patients treated with statins for primary prevention.

## Methods

### Study design and data sources

We conducted a multiregional cohort study, using a distributed network approach with a common data analysis protocol. The pre-specified study protocol has been published [13]. This study is reported as per the Reporting of Studies Conducted using Observational Routinely-Collected Data (RECORD) guideline (S1 Checklist).

We used electronic healthcare databases from three geographical regions to identify eligible patients initiating statins for primary prevention. For the HK cohort, electronic health record data were extracted from the Clinical Data and Analysis Reporting System of the Hong Kong Hospital Authority [14]. The Hospital Authority provides public primary, secondary and tertiary healthcare for Hong Kong’s 7.5 million residents. For the NZ cohort, we used administrative data from the National Health Collections maintained by the Ministry of Health and Health New Zealand. The data used in this study form part of the Medicines Intelligence Research Platform and represents linked, encrypted, de-identified patient-level data comprised of the National Minimum Dataset (public hospital episodes), the Pharmaceutical Collection (community pharmaceutical dispensing claims), the Mortality Register, the Primary Health Organisation National Enrolment Service (primary care registration data and patient demographics), to which a study-specific laboratory results dataset (covering Auckland and Northland regions only) was linked. For the UK cohort, we used anonymised electronic health records from primary care (Clinical Practice Research Datalink [CPRD] GOLD), linked to Hospital Episode Statistics Admitted Patient Care, and Office for National Statistics mortality data. The CPRD data is broadly representative of the UK general population and has been used for many epidemiological studies [15,16]. Ethics approval was obtained from the Institutional Review Board of the University of Hong Kong/Hospital Authority Hong Kong West Cluster (UW 17–135) and from the Independent Scientific Advisory Committee for CPRD (ISAC Protocol 17_200R). The study was reviewed by the New Zealand Health and Disability Ethics Committee and deemed exempt.

### Eligibility criteria

Individuals who were prescribed (UK) or dispensed (HK and NZ) a statin between 1 January 2007 and 31 December 2012 were identified in each database, with the first date of a statin prescription or dispensing defined as the index date. The cohort enrolment window was selected based on the available data sources, to align calendar time across cohorts and to allow sufficient follow-up time to accrue infrequent outcomes in a lower-risk primary prevention population. We then restricted the cohort to individuals newly initiating statins, defined as having no previous lipid-modifying drug prescription during a 12-month washout window prior to the index date and individuals with records marked as having acceptable data quality for use in research (CPRD only).

We excluded patients without baseline lipid tests (minimum of total, LDL and high-density lipoprotein [HDL] cholesterol) within one year of the index date, without at least 12 months of clinical records after registration in a practice (UK only), with a previous diagnosis of ASCVD, heart failure or chronic kidney disease, aged <18 years, <2 additional statin prescriptions within 1 year after the index date and without follow-up lipid tests within 1 year prior to the year 2 landmark. The eligibility criteria were selected to identify the target population of interest: adults at generally low to moderate risk of cardiovascular events who initiated statins for primary prevention.

### Exposures

The landmark method was used to address selection bias caused by classification of patients according to treatment response during follow-up in time-to-event analyses [17]. The landmark method requires selection of a pre-specified fixed time after treatment initiation as the follow-up start time, chosen to allow sufficient time to observe and classify treatment response while minimizing exclusions due to early events. Only patients who were alive and who had not experienced any of the outcomes of interest were therefore included in the analysis. Whether an individual was included in the analysis was conditional on the availability of follow-up lipid tests and survival (without MACE or death) until the landmark. We selected a landmark 2 years after statin initiation to ensure adequate time for clinicians to measure follow-up lipid tests in each database and because this is the estimated time to benefit for statin therapy in primary prevention [18,19].

Categorical cut-points for LDL and non-HDL cholesterol were selected based on specific treatment targets in the 2019 European Society of Cardiology and 2015 National Lipid Association clinical practice guidelines for dyslipidaemia [20,21]. Each individual was classified according to their achieved concentration of LDL (<1.4, 1.4–1.7, 1.8–2.5, ≥2.6 mmol/L) and non-HDL cholesterol (<2.2, 2.2–2.5, 2.6–3.3, ≥3.4 mmol/L) at the landmark. If not reported in the database, non-HDL cholesterol was calculated as total cholesterol – HDL cholesterol. In situations where multiple lipid tests were available for an individual, the latest test result within the assessment window (nearest the landmark) was selected. Statin intensity was defined according to the 2018 American Heart Association/American College of Cardiology guidelines for management of blood cholesterol [22].

### Outcomes

The primary outcome was MACE, defined as the first occurrence of coronary heart disease, stroke or cardiovascular death. Secondary outcomes included the individual components of the composite MACE outcome and all-cause mortality. For each analysis, follow-up began at the landmark time and continued until the earliest occurrence of the outcome of interest, death, disenrollment or transfer from primary care practice or the administrative end of follow-up (31 December 2017).

### Statistical analyses

Descriptive statistics were reported for the included individuals overall and in each database according to categories of achieved LDL and non-HDL cholesterol at the landmark. Categorical variables were reported as numbers (%) and continuous variables as means (standard deviation [SD]), except for triglycerides (median and interquartile range) because of its skewed distribution. Crude incidence rates (per 1,000 person-years) were estimated with exact Poisson 95% confidence intervals (CI). Cox regression was used to estimate hazard ratios (HR) for categories of achieved LDL and non-HDL cholesterol, with the highest concentration category of LDL (≥2.6 mmol/L) or non-HDL cholesterol (≥3.4 mmol/L) defined as the reference level. For all analyses, separate models were fit for LDL and non-HDL cholesterol, and they were not adjusted for each other given that they are highly correlated variables. Cox regression models were adjusted for pre-specified confounding factors as listed in the study protocol: age, sex, hypertension, atrial fibrillation, diabetes mellitus, dyslipidaemia, obesity, smoking, alcohol misuse, rheumatoid arthritis, systemic lupus erythematosus and connective tissue disorders, severe mental illness, antihypertensive drugs, antidiabetic drugs, antiplatelet drugs, and anticoagulants [13]. The proportional hazards assumption was assessed by plotting scaled Schoenfeld residuals. In models assessing the continuous relationships for atherogenic cholesterol, LDL or non-HDL cholesterol were modeled flexibly using penalized regression splines (pspline function in the R *survival* package) with 3 degrees of freedom (continuous relationship pre-specified in published protocol with the specific spline implementation determined during analysis development). Regression splines for achieved LDL and non-HDL cholesterol were used to model adjusted HRs and 95% CI on the log scale for each outcome. Regression splines were centered at the lower bound of the reference categories for LDL cholesterol (2.6 mmol/L) and non-HDL cholesterol (3.4 mmol/L). The categorical analyses based on guideline-defined cut-offs were the main analyses and the continuous analyses were used to describe the overall shape of the association without imposing cut-offs and are presented as complementary analyses. Spline estimates are less precise where data are sparse, hence estimates at the extremes of the distribution were not interpreted. Statistical analyses were run independently at each site following the common protocol. The coordinating site (HK) used a random effects meta-analysis model with the restricted maximum likelihood estimator to pool regression coefficients using the *metafor* package in R (version 4.3.3) [23]. Heterogeneity was assessed using *I*^2^.

### Subgroup and sensitivity analyses

For the primary outcome of MACE, pre-specified subgroup analyses were conducted by age (<70 years, ≥70 years), sex (male, female), statin adherence prior to the landmark (<80%, ≥80% proportion of days covered), persistent with statin at the landmark (yes, no), statin dose intensity at baseline (low, moderate/high), statin dose intensity at the landmark (low, moderate/high) and ethnicity (cohort-specific estimates for HK [Chinese, other ethnicity] and NZ [Māori, non-Māori]). Post-hoc subgroups included diabetes mellitus (yes or no). A pre-specified separate analysis categorized LDL cholesterol by the concentrations examined in the Further Cardiovascular Outcomes Research with PCSK9 Inhibition in Subjects with Elevated Risk (FOURIER) trial (<0.5 mmol/l, 0.5 to <1.3 mmol/l, 1.3 to <1.8 mmol/l, 1.8 to <2.6 mmol/l, and ≥2.6 mmol/l) [24]. To address the limitations of selecting a two-year landmark and improve the generalizability of our results to different time points, we conducted sensitivity analyses using alternative landmarks at 1 year and 3 months after statin initiation. Furthermore, to assess the robustness of our main analysis, we conducted an additional sensitivity analysis using only UK data to adjust for additional confounders that were not available in the other databases. For UK only, the additional confounding variables included in our adjusted models were baseline body mass index (BMI), systolic blood pressure, diastolic blood pressure and family history of cardiovascular disease. We used multiple imputation with chained equations to impute missing continuous variables in 10 imputed datasets, which were pooled using Rubin’s rules.

### Variations from the published study protocol

For the NZ cohort, data from PREDICT database referred to in the study protocol were not accessible, therefore, we obtained data from the NZ Ministry of Health and curated as the National Collections at the University of Auckland. After beginning analyses, data limitations from the CPRD extract did not allow for subgroup analyses for statin adherence and persistence, ethnicity, nor evaluation of sensitivity analyses using a 3-month landmark or LDL cholesterol categories evaluated in FOURIER. After peer review, we conducted an additional sensitivity analysis excluding the NZ cohort.

### Use of AI disclosure

Artificial intelligence tools were not used in this study.

## Results

We included 185,237 patients who initiated statins for primary prevention between 2007 and 2012, with the largest proportion of patients contributing to the analysis from HK, followed by the UK and NZ (Fig 1). The UK had the highest proportion (5.3%) and HK had the lowest proportion (3.8%) of patients excluded because they experienced MACE or all-cause mortality prior to the year 2 landmark. At 2 years after statin initiation, LDL cholesterol was <1.4 mmol/L in 7,084 (3.8%) patients, 1.4–1.7 mmol/L in 21,893 (11.8%) patients, 1.8–2.5 mmol/L in 81,941 (44.2%) patients and ≥2.6 mmol/L in 74,294 (40.1%) patients (Table 1). Overall, the mean age across achieved LDL cholesterol categories was 58–62 years, and there was a modestly higher proportion of men in the two lowest categories. Mean baseline LDL and non-HDL cholesterol were highest in the LDL cholesterol ≥2.6 mmol/L category, while the lower categories had a greater frequency of comorbid cardiovascular risk factors such as hypertension, diabetes mellitus and smoking.

**Table 1:** Baseline characteristics of patients using statins for primary prevention in Hong Kong, New Zealand and the United Kingdom between 2007 and 2012. Figures are the number (%) of patients unless indicated otherwise.

	Overall (*n* = 185,212)			
	Year 2 LDL-C, mmol/L			
**Characteristic**	<1.4	1.4–1.7	1.8–2.5	≥2.6
Number of patients	7,084 (3.8%)	21,893 (11.8%)	81,941 (44.2%)	74,294 (40.1%)
Sex				
Men	4,142 (58.5%)	11,614 (53.0%)	41,490 (50.6%)	36,545 (49.2%)
Women	2,942 (41.5%)	10,279 (47.0%)	40,451 (49.4%)	37,749 (50.8%)
Ethnicity				
Chinese (HK)	2,519 (35.6%)	10,918 (49.9%)	44,546 (54.4%)	34,754 (46.8%)
Other ethnicity (HK)	212 (3.0%)	993 (4.5%)	4,222 (5.2%)	3,247 (4.4%)
Māori (NZ)	113 (1.6%)	1,072 (4.9%)	959 (1.2%)	1,228 (1.7%)
Pacific (NZ)	206 (2.9%)	257 (1.2%)	1,485 (1.8%)	1,791 (2.4%)
All Asian (NZ)	342 (4.8%)	482 (2.2%)	3,339 (4.1%)	3,342 (4.5%)
European and other (NZ)	752 (10.6%)	2079 (9.5%)	9,277 (11.3%)	11,170 (15.0%)
Asian/Asian British/other Asian (UK)	46 (0.6%)	70 (0.3%)	216 (0.3%)	216 (0.3%)
Black/African/Caribbean (UK)	28 (0.4%)	40 (0.2%)	110 (0.1%)	166 (0.2%)
Mixed/multiple (UK)	68 (1.0%)	86 (0.4%)	228 (0.3%)	206 (0.3%)
Other ethnic group (UK)	23 (0.3%)	47 (0.2%)	118 (0.1%)	138 (0.2%)
White (UK)	719 (10.1%)	1,649 (7.5%)	4,961 (6.1%)	5,200 (7.0%)
Unknown (UK)	2056 (29.0%)	4,200 (19.2%)	12,480 (15.2%)	12,836 (17.3%)
Age, years (mean (SD))	62.3 (11.8)	62.6 (11.2)	61.4 (10.9)	58.7 (11.1)
Body mass index (mean (SD))*	30.0 (6.0)	29.7 (6.1)	29.2 (5.7)	29.0 (5.4)
Systolic blood pressure (mean (SD))*	140.0 (19.0)	140.0 (18.0)	140.0 (18.0)	139.0 (18.0)
Diastolic blood pressure (mean (SD))*	82.0 (11.0)	82.0 (10.0)	82.0 (11.0)	83.0 (10.0)
**Lipid tests at time of statin initiation**				
Total cholesterol, mmol/L (mean (SD))	5.1 (1.1)	5.5 (1.0)	5.9 (1.0)	6.5 (1.1)
LDL-C, mmol/L (mean (SD))	2.9 (0.9)	3.3 (0.8)	3.8 (1.0)	4.4 (1.0)
LDL-C group, mmol/L (number (%))				
<1.4	429 (6.1%)	293 (1.3%)	217 (0.3%)	69 (0.1%)
1.4–1.7	424 (6.0%)	672 (3.1%)	906 (1.1%)	213 (0.3%)
1.8–2.5	1,323 (18.7%)	2010 (9.2%)	4,610 (5.6%)	2086 (2.8%)
≥2.6	4,908 (69.3%)	18,918 (86.4%)	76,208 (93.0%)	71,926 (96.8%)
non-HDL-C, mmol/L (mean (SD))	3.8 (1.1)	4.1 (0.9)	4.5 (1.0)	5.1 (1.1)
non-HDL-C group, mmol/L (number (%))				
<2.2	524 (7.4%)	599 (2.7%)	696 (0.8%)	222 (0.3%)
2.2–2.5	339 (4.8%)	678 (3.1%)	1,324 (1.6%)	409 (0.6%)
2.6–3.3	1,267 (17.9%)	2,632 (12.0%)	5,771 (7.0%)	2,699 (3.6%)
≥3.4	4,954 (69.9%)	17,984 (82.1%)	74,150 (90.5%)	70,964 (95.5%)
HDL-C, mmol/L (mean (SD))	1.3 (0.4)	1.3 (0.4)	1.3 (0.4)	1.3 (0.4)
**Comorbidities**				
Hypertension	3,220 (45.5%)	10,328 (47.2%)	36,118 (44.1%)	26,743 (36.0%)
Atrial fibrillation	183 (2.6%)	401 (1.8%)	1,427 (1.7%)	1,069 (1.4%)
Diabetes mellitus	3,924 (55.4%)	11,886 (54.3%)	35,810 (43.7%)	19,522 (26.3%)
Mental health disorder	403 (5.7%)	1,230 (5.6%)	4,784 (5.8%)	4,613 (6.2%)
Obesity	284 (4.0%)	604 (2.8%)	1,611 (2.0%)	1,667 (2.2%)
Dyslipidemia	471 (6.6%)	1,578 (7.2%)	6,960 (8.5%)	8,856 (11.9%)
Smoking	919 (13.0%)	1921 (8.8%)	5,822 (7.1%)	6,578 (8.9%)
COPD#	74 (1.0%)	255 (1.2%)	850 (1.0%)	776 (1.0%)
Chronic liver disease	117 (1.7%)	355 (1.6%)	1,367 (1.7%)	1,078 (1.5%)
Chronic pancreatitis	17 (0.2%)	41 (0.2%)	83 (0.1%)	70 (0.1%)
Rheumatoid arthritis and related disorders	31 (0.4%)	82 (0.4%)	335 (0.4%)	434 (0.6%)
SLE	16 (0.2%)	33 (0.2%)	189 (0.2%)	214 (0.3%)
Alcohol misuse	123 (1.7%)	171 (0.8%)	775 (0.9%)	825 (1.1%)
History of substance use^#^	47 (0.7%)	95 (0.4%)	385 (0.5%)	341 (0.5%)
**Medications**				
Statin drug				
Atorvastatin	755 (10.7%)	1886 (8.6%)	5,826 (7.1%)	5,769 (7.8%)
Fluvastatin	9 (0.1%)	18 (0.1%)	88 (0.1%)	140 (0.2%)
Lovastatin	0 (0.0%)	0 (0.0%)	1 (0.0%)	0 (0.0%)
Pravastatin	53 (0.7%)	105 (0.5%)	322 (0.4%)	531 (0.7%)
Rosuvastatin	864 (12.2%)	1814 (8.3%)	5,399 (6.6%)	5,477 (7.4%)
Simvastatin	5,403 (76.3%)	18,070 (82.5%)	70,305 (85.8%)	62,377 (84.0%)
Statin intensity				
Low	2,466 (34.8%)	10,714 (48.9%)	44,956 (54.9%)	35,972 (48.4%)
Moderate	4,303 (60.7%)	10,554 (48.2%)	35,656 (43.5%)	37,082 (49.9%)
High	315 (4.4%)	625 (2.9%)	1,329 (1.6%)	1,240 (1.7%)
Other lipid-lowering drugs	43 (0.6%)	93 (0.4%)	283 (0.3%)	507 (0.7%)
Diabetes drug	3,662 (51.7%)	11,289 (51.6%)	34,317 (41.9%)	18,352 (24.7%)
Insulin^#^	282 (4.0%)	933 (4.3%)	2,788 (3.4%)	1802 (2.4%)
Non-insulin diabetes drug#	2,376 (33.5%)	9,398 (42.9%)	30,843 (37.6%)	16,022 (21.6%)
Aspirin^#^	719 (10.1%)	2,223 (10.2%)	7,261 (8.9%)	4,900 (6.6%)
Other antiplatelet drugs (All antiplatelet drugs in UK)	954 (13.5%)	1,657 (7.6%)	3,617 (4.4%)	2,513 (3.4%)
Oral anticoagulants	135 (1.9%)	304 (1.4%)	895 (1.1%)	685 (0.9%)
Antihypertensives	4,335 (61.2%)	14,202 (64.9%)	50,954 (62.2%)	38,658 (52.0%)

Abbreviations: COPD, chronic obstructive pulmonary disease; HDL-C, high-density lipoprotein cholesterol; HK, Hong Kong; IQR, interquartile range; LDL-C, low-density lipoprotein cholesterol; non-HDL-C, non-high-density lipoprotein cholesterol; NZ, New Zealand; SD, standard deviation; SLE, systemic lupus erythematosus and connective tissue disorders; TC, total cholesterol; UK, United Kingdom.

*Variable available for UK cohort.

# Variable available in HK and NZ cohorts.

**Fig 1:**
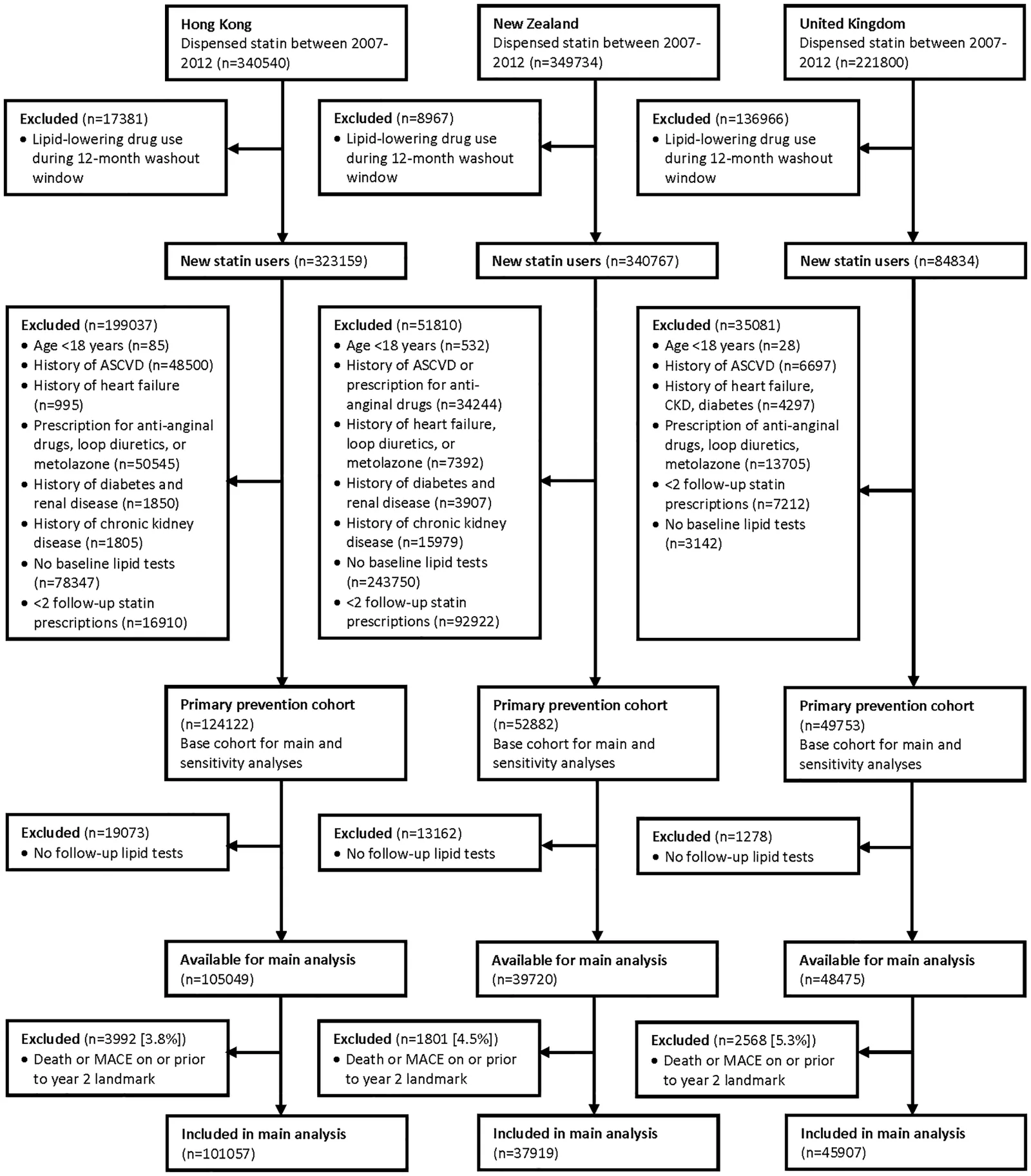
Flowchart of patients and application of eligibility criteria in the study cohorts. Abbreviations: ASCVD, atherosclerotic cardiovascular disease; MACE, major adverse cardiovascular events.

Simvastatin was consistently the most prescribed statin, followed by atorvastatin and rosuvastatin (Table 1). Across cohorts, atorvastatin was more often prescribed in the UK and NZ than in HK, and there was no use of rosuvastatin in NZ (Table A in S1 Text). Overall, most patients initiated a moderate-intensity statin, although low-intensity statins were used more frequently in the HK and NZ cohorts as compared with the UK. Less than 1% of patients overall used a non-statin lipid-lowering drug at baseline.

### Association with major adverse cardiovascular events

The mean (SD) duration of follow-up for the primary outcome of MACE was 6.5 (1.5) years in HK, 5.6 (1.9) years in NZ and 5.4 (2.4) years in the UK, resulting in a total of 1,051,128 person-years of observation time. The total number of MACE by achieved LDL cholesterol category were 434 (at <1.4 mmol/L), 1,257 (at 1.4–1.7 mmol/L), 4,528 (at 1.8–2.5 mmol/L) and 44,298 (at ≥2.6 mmol/L; Table 2). Crude incidence rates across cohorts were similar for MACE for the 1.8–2.5 mmol/L and ≥2.6 mmol/L LDL cholesterol categories but were much higher in NZ as compared to HK and the UK for the <1.4 mmol/L and 1.4–1.7 mmol/L categories (Table B in S1 Text).

**Table 2:** Total number of events, patients and follow-up time, and pooled hazard ratios for major adverse cardiovascular events and the individual component outcomes, and all-cause mortality, stratified by achieved LDL and non-HDL cholesterol category at the year 2 landmark.

Outcome		No. of events	No. of patients	No. of p-y	Crude HR (95% CI)	I^2^ (%)	Adjusted HR (95% CI)	I^2^ (%)
**LDL cholesterol, mmol/L**								
Major adverse cardiovascular events								
	<1.4	434	7,084	36,999	1.04 (0.74,1.46)	89	0.82 (0.60,1.12)	90
	1.4–1.7	1,257	21,893	120,925	0.98 (0.88,1.09)	53	0.79 (0.66,0.94)	87
	1.8–2.5	4,529	81,941	468,640	0.93 (0.84,1.03)	76	0.80 (0.77,0.83)	0
	≥2.6	4,298	74,294	424,564	1.00		1.00	
Cardiovascular death								
	<1.4	60	7,084	37,371	1.20 (0.89,1.63)	0	0.75 (0.47,1.19)	67
	1.4–1.7	208	21,893	122,314	1.28 (0.97,1.67)	46	0.93 (0.59,1.44)	85
	1.8–2.5	537	81,941	474,418	1.01 (0.89,1.14)	0	0.78 (0.69,0.87)	0
	≥2.6	502	74,294	430,031	1.00		1.00	
Coronary heart disease								
	<1.4	258	7,083	37,132	0.96 (0.66,1.39)	84	0.79 (0.58,1.08)	84
	1.4–1.7	716	21,899	121,386	0.87 (0.67,1.12)	84	0.74 (0.60,0.92)	85
	1.8–2.5	2,628	81,953	470,829	0.85 (0.67,1.06)	91	0.76 (0.69,0.83)	65
	≥2.6	2,662	74,302	426,440	1.00		1.00	
Stroke								
	<1.4	152	7,084	37,229	1.11 (0.82,1.51)	62	0.82 (0.66,1.03)	46
	1.4–1.7	481	21,893	121,824	1.08 (0.90,1.29)	55	0.87 (0.66,1.15)	86
	1.8–2.5	1800	81,941	472,064	1.04 (0.95,1.14)	28	0.88 (0.79,0.97)	57
	≥2.6	1,537	74,294	427,951	1.00		1.00	
All-cause mortality								
	<1.4	479	7,084	37,371	1.85 (1.66,2.05)	0	1.06 (0.96,1.16)	0
	1.4–1.7	1,251	21,893	122,314	1.41 (1.31,1.51)	0	1.01 (0.83,1.22)	87
	1.8–2.5	3,769	81,941	474,418	1.10 (0.98,1.23)	67	0.89 (0.79,1.00)	81
	≥2.6	3,125	74,294	430,031	1.00		1.00	
**non-HDL cholesterol, mmol/L**								
Major adverse cardiovascular events								
	<2.2	1,128	19,247	103,762	0.95 (0.79,1.14)	80	0.73 (0.57,0.93)	92
	2.2–2.5	1,695	30,833	173,280	0.91 (0.85,0.97)	0	0.72 (0.63,0.81)	78
	2.6–3.3	3,782	70,268	403,056	0.87 (0.79,0.96)	72	0.75 (0.72,0.79)	0
	≥3.4	3,913	64,864	371,030	1.00		1.00	
Cardiovascular death								
	<2.2	151	19,247	104,973	1.12 (0.81,1.56)	53	0.70 (0.46,1.05)	80
	2.2–2.5	247	30,833	175,303	1.16 (0.99,1.36)	0	0.74 (0.64,0.87)	0
	2.6–3.3	456	70,268	407,968	0.87 (0.69,1.10)	54	0.75 (0.55,1.01)	80
	≥3.4	453	64,864	375,891	1.00		1.00	
Coronary heart disease								
	<2.2	638	19,247	104,197	0.81 (0.59,1.13)	88	0.67 (0.51,0.87)	89
	2.2–2.5	970	30,833	174,048	0.80 (0.68,0.94)	65	0.67 (0.57,0.79)	77
	2.6–3.3	2,209	70,268	404,875	0.80 (0.65,0.97)	88	0.71 (0.66,0.77)	38
	≥3.4	2,447	64,864	372,666	1.00		1.00	
Stroke								
	<2.2	457	19,247	104,499	1.15 (1.03,1.29)	0	0.85 (0.66,1.08)	80
	2.2–2.5	658	30,833	174,487	1.02 (0.86,1.21)	57	0.78 (0.64,0.93)	72
	2.6–3.3	1,486	70,268	406,041	0.99 (0.86,1.14)	60	0.85 (0.74,0.98)	70
	≥3.4	1,369	64,864	374,041	1.00		1.00	
All-cause mortality								
	<2.2	1,204	19,247	104,973	1.58 (1.47,1.70)	0	0.98 (0.84,1.14)	78
	2.2–2.5	1,627	30,833	175,303	1.38 (0.98,1.95)	95	0.93 (0.75,1.17)	92
	2.6–3.3	3,075	70,268	407,968	1.05 (0.93,1.19)	69	0.85 (0.76,0.95)	78
	≥3.4	2,718	64,864	375,891	1.00		1.00	

Abbreviations: CI, confidence interval; HR, hazard ratio; LDL, low-density lipoprotein; non-HDL, non-high-density lipoprotein; p-y, person-year.

As compared to LDL cholesterol ≥2.6 mmol/L, achieving a concentration of 1.8–2.5 mmol/L was associated with a reduced risk of MACE with low heterogeneity (adjusted HR 0.80, 95% CI [0.77,0.83]; Table 2). Adjusted HRs for the <1.4 mmol/L and 1.4–1.7 mmol/L categories were also associated with a lower risk of MACE, notably in the HK and UK cohorts, but there was higher heterogeneity because of the estimates from the NZ cohort, which included the null value (Fig A in S1 Text). The pooled estimates for cardiovascular death and coronary heart disease were similar to those obtained for MACE, while estimates for stroke were more attenuated, with the data being consistent with an associated 12% risk reduction for the 1.8–2.5 mmol/L LDL cholesterol category.

The number of MACE by achieved non-HDL cholesterol was 1,128 (at <2.2 mmol/L), 1,695 (2.2–2.5 mmol/L), 3,782 (2.6–3.3 mmol/L) and 3,913 (at ≥3.4 mmol/L; Table 2). Crude incidence rates across cohorts were similar for MACE for categories of non-HDL cholesterol but were much higher in NZ as compared to HK and the UK for the <2.2 mmol/L and 2.2–2.5 mmol/L categories (Table B in S1 Text).

Non-HDL cholesterol was more strongly associated with reductions in MACE in both crude and adjusted analyses as compared to the corresponding LDL cholesterol categories. As compared to a non-HDL cholesterol ≥3.4 mmol/L, achieving a non-HDL cholesterol of 2.6–3.3 mmol/L was associated with a reduced risk of MACE (adjusted HR 0.75, 95% CI [0.72,0.79]; Table 2). Pooled HRs for the <2.2 mmol/L and 2.2–2.5 mmol/L categories were also associated with a lower risk of MACE, but there was higher heterogeneity in the <2.2 mmol/L category as a result of the NZ estimate, which included the null value (Fig B in S1 Text).

The continuous relationship for MACE across the full spectrum of on-treatment levels of LDL and non-HDL cholesterol was log-linear in the HK and UK cohorts, whereas in the NZ cohort risk was higher at the lower concentrations (Fig 2). In HK and the UK, the lowest concentrations of LDL and non-HDL cholesterol were therefore associated with the lowest adjusted HRs for MACE after adjusting for confounding factors. In NZ, a positive gradient above approximately 2.5 mmol/L LDL cholesterol was evident. The divergence from the other cohorts was confined to the lowest concentrations and to the sparsely populated upper tail of the distribution, where confidence intervals were wide. The shape of the continuous relationship for MACE was generally consistent in sensitivity analyses using earlier landmarks and varying the degrees of freedom.

**Fig 2:**
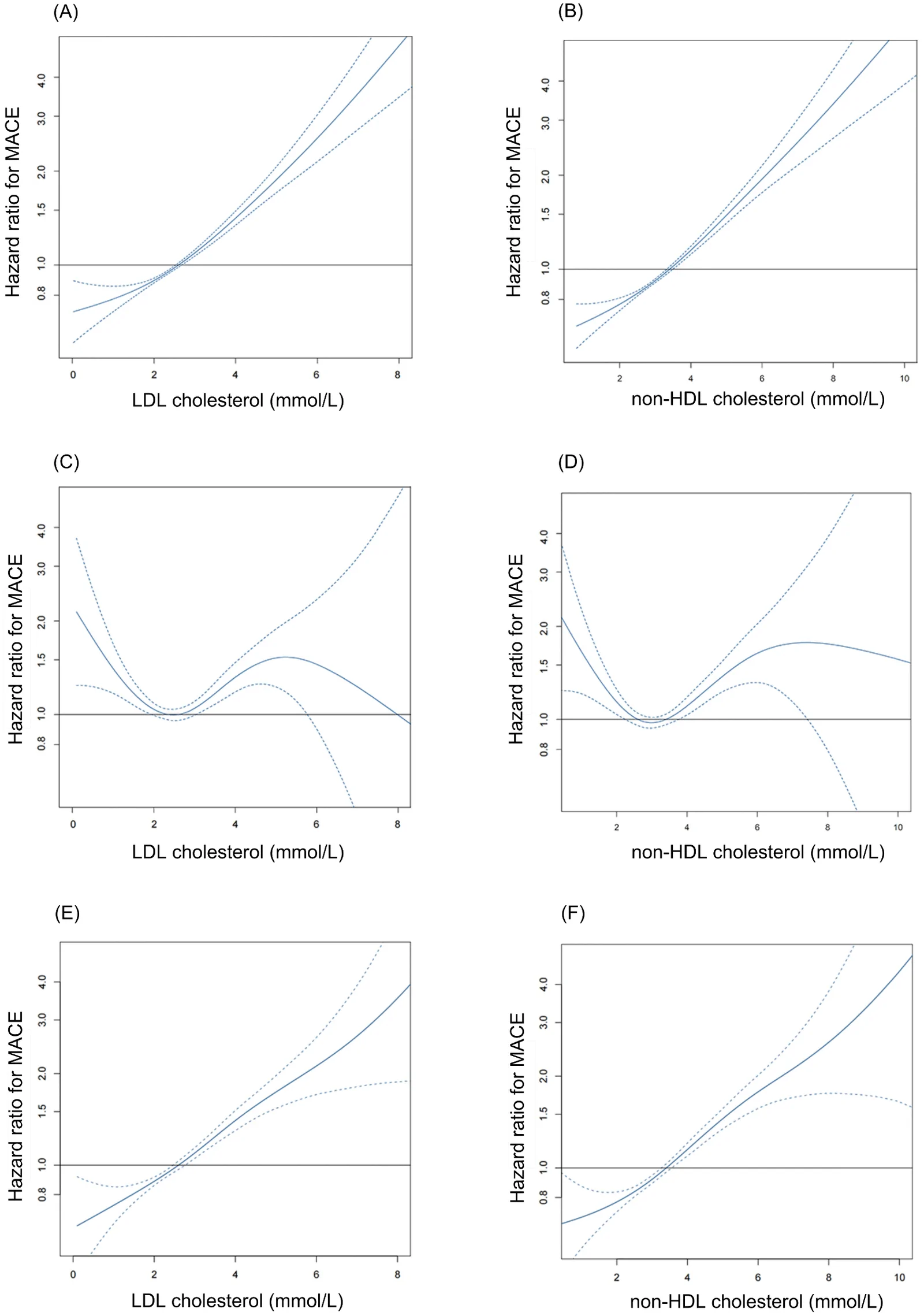
Continuous association for on-treatment low-density lipoprotein (LDL) cholesterol and non–high-density lipoprotein (non-HDL) cholesterol and major adverse cardiovascular events (MACE). Solid blue lines are the multivariable-adjusted hazard ratios (HR), dashed lines show 95% confidence intervals and the horizontal black line indicates the reference line of no association (HR = 1.0). HRs were centered at LDL cholesterol of 2.6 mmol/L or non-HDL cholesterol of 3.4 mmol/L and are plotted on the log scale. Panels show Hong Kong for LDL cholesterol **(A)** and non-HDL cholesterol **(B)**, New Zealand for LDL cholesterol **(C)** and non-HDL cholesterol **(D)**, and United Kingdom for LDL cholesterol **(E)** and non-HDL cholesterol **(F). Abbreviations:** LDL, low-density lipoprotein; MACE, major adverse cardiovascular events; non-HDL, non-high-density lipoprotein.

### Association with all-cause mortality

The number of all-cause deaths by achieved LDL cholesterol was 479 (at <1.4 mmol/L), 1,251 (1.4–1.7 mmol/L), 3,769 (1.8–2.5 mmol/L) and 3,125 (at ≥2.6 mmol/L; Table 2). Crude incidence rates across cohorts for all-cause mortality varied across cohorts, with HK having the highest in the ≥2.6 mmol/L LDL cholesterol category and NZ and HK having higher incidence rates than the UK in the <1.4 mmol/L category (Table B in S1 Text).

Before adjustment for confounding factors, LDL cholesterol <1.4 mmol/L (crude HR 1.85, 95% CI [1.66,2.05]) and 1.4–1.7 mmol/L (crude HR 1.41, 95% CI [1.31,1.51]) were both strongly associated with an increase in all-cause mortality (Table 2). However, in the adjusted models, this association was completely attenuated with no clear association with either an increase or decrease in all-cause mortality for all categories of LDL cholesterol (Fig C in S1 Text). Similarly, the two lowest groups of achieved non-HDL cholesterol were not associated with either an increase or decrease in all-cause mortality after adjustment for confounding factors, but an achieved non-HDL cholesterol of 2.6–3.3 mmol/L was associated with a reduction in all-cause mortality (adjusted HR 0.85, 95% CI [0.76,0.95]; Table C and Fig D in S1 Text).

The continuous relationship for all-cause mortality across the full spectrum of on-treatment levels of LDL and non-HDL cholesterol was less consistent across cohorts (Fig 3). In the NZ cohort, the lowest concentrations of LDL cholesterol were associated with an increased risk of mortality, but the relationship was more J-shaped in the HK and UK cohorts. An achieved LDL cholesterol of 2.0 mmol/L in HK, 2.8 mmol/L in NZ, and 2.8 mmol/L in the UK (within the range of 1–5 mmol/L) was associated with the lowest risk of all-cause mortality. The non-HDL cholesterol relationship indicated a threshold of benefit in the HK and UK cohorts but was U-shaped in NZ. An achieved non-HDL cholesterol of 2.5 mmol/L in HK, 3.3 mmol/L in NZ, and 3.9 mmol/L in the UK (within the range of 1.8–5.8 mmol/L) was associated with the lowest risk of all-cause mortality. The shape of the continuous relationship for all-cause mortality was generally consistent in sensitivity analyses using earlier landmarks and varying the degrees of freedom.

**Fig 3:**
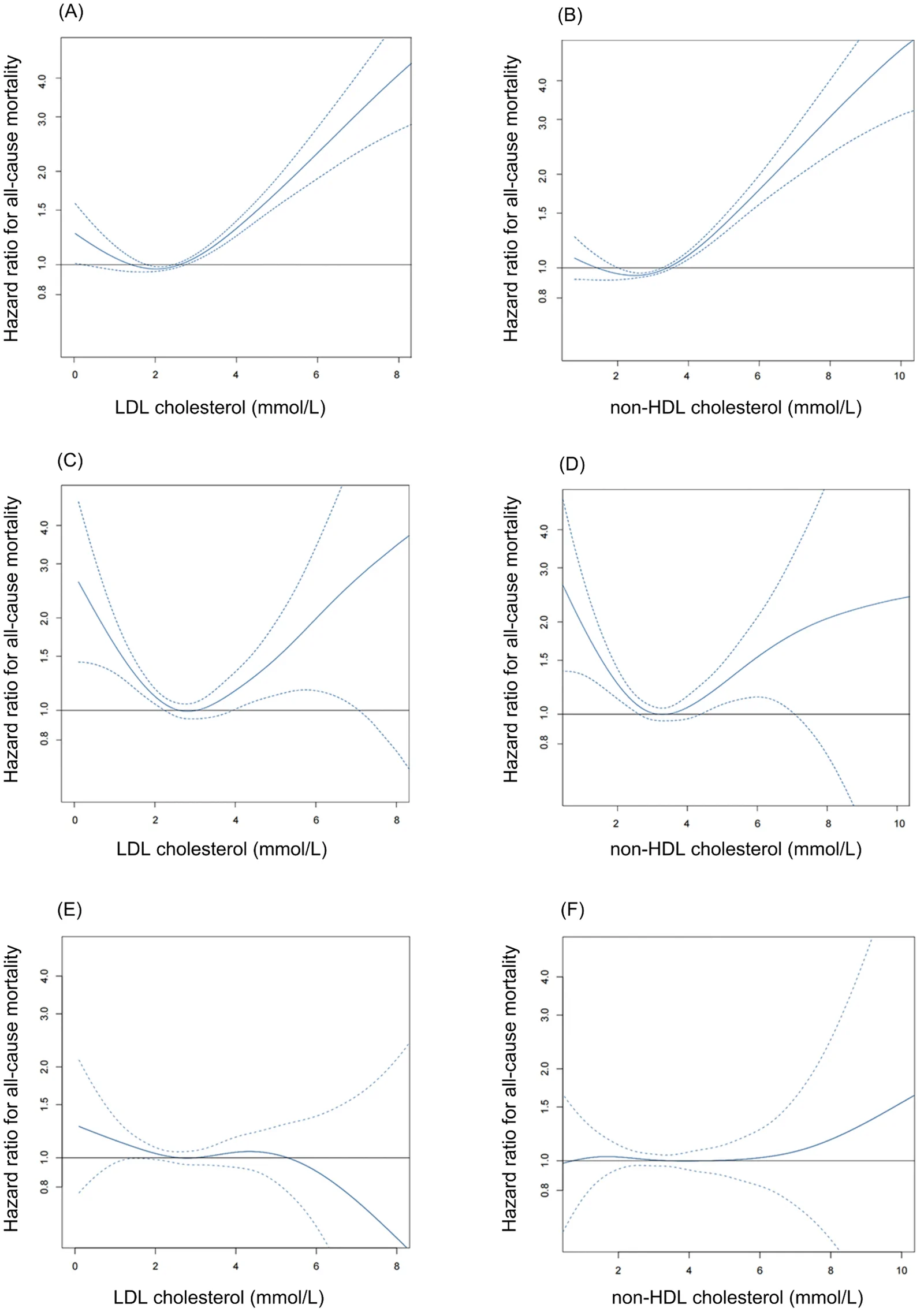
Continuous association for on-treatment low-density lipoprotein (LDL) cholesterol and non–high-density lipoprotein (non-HDL) cholesterol and all-cause mortality. Solid blue lines are the multivariable-adjusted hazard ratios (HR), dashed lines show 95% confidence intervals and the horizontal black line indicates the reference line of no association (HR = 1.0). HRs were centered at LDL cholesterol of 2.6 mmol/L or non-HDL cholesterol of 3.4 mmol/L and are plotted on the log scale. Panels show Hong Kong for LDL cholesterol **(A)** and non-HDL cholesterol **(B)**, New Zealand for LDL cholesterol **(C)** and non-HDL cholesterol **(D)**, and the United Kingdom for LDL cholesterol **(E)** and non-HDL cholesterol **(F). Abbreviations:** LDL, low-density lipoprotein; non-HDL, non-high-density lipoprotein.

### Subgroup and sensitivity analyses

Subgroup analyses demonstrated generally consistent estimates with the main analyses, with similar estimates regardless of age, sex, statin adherence, ethnic group, baseline LDL and non-HDL cholesterol and statin intensity. However, patients with diabetes consistently demonstrated a larger associated reduction in MACE with low heterogeneity across cohorts for achieved levels of LDL and non-HDL cholesterol (Table 3 and Table C in S1 Text). In sensitivity analyses using a 1-year landmark, associations for LDL cholesterol were directionally consistent with the main analysis for MACE and all-cause mortality, supporting the robustness of our findings, but were more imprecise and included the null in the <1.4 mmol/L LDL cholesterol group for cardiovascular death and stroke (Table D in S1 Text). In contrast to the main analysis, achieving an LDL cholesterol of 1.4–1.7 or 1.8–2.5 mmol/L at 3 months was associated with a reduction in all-cause mortality. Associations for non-HDL cholesterol and MACE were more consistent at the 3-month and 1-year landmarks and were also associated with reductions in all-cause mortality for all categories of non-HDL cholesterol with low heterogeneity (Table E in S1 Text). In additional analysis of the UK cohort, estimates were unchanged after additional adjustment for clinical variables that included BMI, systolic blood pressure, diastolic blood pressure and family history of cardiovascular disease (Table F in S1 Text). When LDL cholesterol categories were redefined as in the FOURIER trial (HK and NZ only), there was insufficient data to generate estimates for <0.5 mmol/L and the estimates for 0.5–1.2 mmol/L were highly imprecise for the outcome of MACE (Table G in S1 Text). For 0.5–1.2 mmol/L, the adjusted HR for all-cause mortality was 1.36 (95% CI 0.99,1.87) although because of substantial heterogeneity these findings should also be considered inconclusive rather than null (Table G in S1 Text). After excluding the NZ cohort, estimates for the lowest LDL and non-HDL cholesterol categories shifted towards greater benefit and heterogeneity was reduced for MACE; estimates for other categories and for all all-cause mortality were similar as the primary analysis (Table H in S1 Text).

**Table 3:** Results of subgroup analyses for major adverse cardiovascular events by LDL cholesterol category at the year 2 landmark.

		Adjusted hazard ratio (95% CI); *I*^2^ (%)					
LDL cholesterol, mmol/L		<1.4 vs. ≥2.6 mmol/L	*I*^2^ (%)	1.4–1.7 vs. ≥2.6	*I*^2^ (%)	1.8–2.5 vs. ≥2.6 mmol/L	*I*^2^ (%)
Main analysis		0.85 (0.58,1.26)	90.6	0.80 (0.64,1.01)	86.8	0.81 (0.75,0.88)	58.6
Age (years)							
	≥70	0.85 (0.51,1.41)	87.0	0.81 (0.61,1.09)	78.9	0.86 (0.80,0.92)	0
	<70	0.83 (0.55,1.27)	85.8	0.79 (0.65,0.97)	75.4	0.78 (0.72,0.85)	53
Sex							
	Women	0.85 (0.59,1.22)	70.5	0.79 (0.66,0.96)	58.3	0.86 (0.75,0.99)	67.3
	Men	0.82 (0.54,1.26)	88.5	0.79 (0.64,0.98)	77.9	0.78 (0.74,0.82)	0
Statin adherence, within 2 years after statin initiation*							
	≥80% PDC	1.03 (0.63,1.69)	91.4	0.91 (0.59,1.39)	94.7	0.87 (0.77,0.98)	70.8
	<80% PDC	1.10 (0.66,1.85)	23.5	0.60 (0.23,1.56)	64.4	0.75 (0.64,0.88)	0
Statin persistence, 2 years after statin initiation*							
	Persistent	1.02 (0.64,1.62)	90.3	0.87 (0.61,1.24)	92	0.86 (0.76,0.96)	65.1
	Not persistent	0.92 (0.60,1.41)	0	0.97 (0.69,1.36)	34	0.84 (0.72,0.98)	0
Ethnic group (HK only)							
	Chinese	0.81 (0.70,0.94)	--	0.72 (0.66,0.79)	--	0.81 (0.76,0.85)	--
	Other ethnicity	0.44 (0.16,1.21)	--	0.67 (0.43,1.05)	--	0.63 (0.47,0.84)	--
Ethnic group (NZ only)							
	Māori	0.56 (0.13,2.33)	--	1.21 (0.61,2.41)	--	1.27 (0.83,1.94)	--
	non-Māori	1.28 (1.01,1.62)	--	1.03 (0.86,1.22)	--	0.87 (0.78,0.98)	--
Statin intensity, index date							
	Low	0.84 (0.71,0.99)	0	0.77 (0.63,0.96)	40.5	0.82 (0.77,0.87)	0
	Moderate or high	0.97 (0.46,2.05)	93.6	0.84 (0.55,1.30)	90.8	0.82 (0.69,0.97)	73.5
Statin intensity, year 2 landmark							
	Low	0.86 (0.73,1.02)	0	0.93 (0.54,1.58)	85.4	0.90 (0.71, 1.14)	67.2
	Moderate or high	0.99 (0.54,1.80)	90.3	0.87 (0.64,1.17)	83.3	0.85 (0.77, 0.94)	39.2
Diabetes mellitus							
	Yes	0.69 (0.54,0.88)	47.6	0.67 (0.61,0.73)	0	0.75 (0.70, 0.80)	0
	No	0.95 (0.64,1.40)	86.5	0.87 (0.71,1.06)	77.2	0.85 (0.77, 0.93)	66.1
Baseline total cholesterol, mmol/L							
	≥5.8	0.81 (0.62,1.06)	0	0.75 (0.65,0.86)	0	0.84 (0.77, 0.92)	29.7
	<5.8	0.98 (0.71,1.34)	76.2	0.84 (0.67,1.04)	72.9	0.82 (0.76, 0.88)	0
Baseline LDL cholesterol, mmol/L							
	≥3.8	1.07 (0.79,1.45)	0	0.84 (0.64,1.10)	47.2	0.83 (0.78, 0.89)	0
	<3.8	0.93 (0.67,1.31)	79.3	0.81 (0.66,1.00)	70	0.81 (0.75, 0.88)	0
Baseline non-HDL cholesterol, mmol/L							
	≥4.5	0.91 (0.71,1.18)	0	0.75 (0.65,0.86)	0	0.83 (0.78, 0.89)	0
	<4.5	0.98 (0.70,1.37)	78.5	0.86 (0.68,1.09)	77	0.83 (0.77, 0.90)	0
						

Abbreviations: CI, confidence interval; HK, Hong Kong; HR, hazard ratio; LDL, low-density lipoprotein; non-HDL, non-high-density lipoprotein; NZ, New Zealand; PDC, proportion of days covered.

*Excludes UK cohort.

## Discussion

In this cohort study of 185,237 new statin users, achieving a non-HDL cholesterol <3.4 mmol/L or an LDL cholesterol <2.6 mmol/L was associated with reductions in MACE of approximately 20%–25%. The most robust estimates, with the highest precision and lowest heterogeneity, were obtained for patients with a non-HDL cholesterol of 2.6–3.3 mmol/L and an LDL cholesterol of 1.8–2.5 mmol/L. In the context of the latest American and European guideline recommendations for management of dyslipidaemia in primary prevention, nearly 40% of patients in our study experienced suboptimal absolute reductions in atherogenic cholesterol because they did not achieve at least a non-HDL cholesterol <3.4 mmol/L or an LDL cholesterol <2.6 mmol/L within 2 years after starting a statin [25,26]. After adjustments for a wide range of measured confounding factors, there was limited evidence of a relationship between very low LDL cholesterol and all-cause mortality with a threshold effect between 2.5 and 3.9 mmol/L in two of the three cohorts. By evaluating the full spectrum of on-treatment atherogenic cholesterol concentrations in diverse populations, this study has important implications for clinicians, patients, and guideline developers seeking to understand the long-term effects of achieving very-low atherogenic cholesterol with statin therapy for primary prevention of ASCVD.

The magnitude of association for LDL and non-HDL cholesterol against MACE is consistent with previous reports that have employed similar analytic approaches to clinical trial and real-world data. The relationship for achieved LDL and non-HDL cholesterol has been examined in two meta-analyses of predominantly secondary prevention statin trials, with a stronger association against MACE for non-HDL cholesterol than LDL cholesterol, a finding that aligns with our study [7,8]. Although not directly comparable because of different categorical definitions for each measure of atherogenic cholesterol, our findings suggest less benefit of achieving very low concentrations of LDL and non-HDL cholesterol when compared to these meta-analyses. Higher concentrations of non-HDL cholesterol, but not LDL cholesterol, were also associated with myocardial infarction in 13,015 statin-treated patients (39% with established ASCVD) in the Copenhagen General Population study [11]. By contrast, we found a consistent log-linear relationship for both LDL and non-HDL cholesterol in the HK and UK cohorts with more precise estimates because of the larger study size. Observational studies of patients with type 2 diabetes mellitus using statins for primary prevention have also shown that achieving very low levels of LDL and non-HDL cholesterol are associated with reductions in cardiovascular events [27,28]. This consistent association for a reduced risk of MACE was observed in our subgroup analysis of patients with diabetes.

Only a few observational studies have evaluated the association between statin-treated LDL and non-HDL cholesterol concentrations and all-cause mortality. In an early cohort study of simvastatin conducted in Japan, achieved LDL cholesterol <2.1 mmol/L (<80 mg/dL) was associated with an increased risk of mortality versus 3.1–3.6 (120–139 mg/dL) [9]. Among statin-treated individuals without ASCVD (*n* = 5,140) in the Copenhagen general population study, an LDL cholesterol of 2.6 mmol/L (101 mg/dL) was associated with the lowest risk of mortality [10,11]. After adjusting for a wide range of confounding factors, the continuous associations in our study demonstrated a threshold of benefit with a modest J-shaped curve for LDL and non-HDL cholesterol. Although these three observational studies evaluated associations with all-cause mortality, our work implies that their findings may be subject to bias because of inadequate control of confounders and selection bias, as a result of inclusion of prevalent users of statins at cohort entry and no consistent timing of lipid assessment after statin initiation.

To our knowledge, this is the largest and most geographically diverse study to evaluate the association between guideline-recommended targets for LDL and non-HDL cholesterol levels among statin-treated patients for primary prevention of ASCVD. To minimize selection biases, we adopted both a new-user and landmark approach, analogous to prior analyses of clinical trial data [7,8]. By assessing MACE, its component outcomes and all-cause mortality, we provide evidence on the clinical outcomes that are most important to patients and clinicians. We pre-specified and published our study protocol before starting data analysis which enhances the transparency of our work. Lastly, because of the global variations in prescribed statin drugs, doses, and individual atherogenic cholesterol-lowering response, our findings are more generalizable because we pooled large-scale data from regions in Asia, Oceania and Europe.

Given the widespread use of statins in primary prevention, this study has several key implications for clinicians. The findings for all-cause mortality are especially important to reassure patients who may have concerns about achieving very low atherogenic cholesterol on the basis of concerning findings reported in prior research which may be amplified in online discourse [29]. The results of this study can be used by clinicians to show patients why consistent use of statins with the goal to achieve lower absolute atherogenic cholesterol levels is essential for the optimal long-term prevention of ASCVD events. Our study suggests that at low concentrations, non-HDL cholesterol is more closely associated with the on-treatment risk of MACE than LDL cholesterol. This is not surprising since non-HDL cholesterol captures the cholesterol content of all apolipoprotein B (apoB)-containing lipoproteins, including LDL, very-low density lipoprotein, intermediate-density lipoprotein, and lipoprotein(a), and therefore correlates closely with direct apoB measurement (correlation coefficient >0.90) [30]. Non-HDL cholesterol and apoB are more strongly associated with cardiovascular events than LDL cholesterol among participants in statin trials and in epidemiological studies [8,31]. The stronger associations for MACE observed for non-HDL compared with LDL cholesterol in our study are consistent with the superiority of non-HDL cholesterol and apoB as measures of residual cardiovascular risk demonstrated in previous meta-analyses [8,31]. LDL cholesterol is most often calculated by clinical laboratories using the Friedewald formula, and has known limitations among patients with elevated triglycerides or very low LDL cholesterol levels [32]. By contrast, non-HDL cholesterol is a more practical measure and was also associated with a 15% reduction in all-cause mortality at lower concentrations (i.e., 2.6–3.3 mmol/L) in our study. In clinical practice, non-HDL cholesterol is recommended as a practical surrogate for apoB when it is unavailable. The ability to calculate non-HDL cholesterol from routinely collected total and HDL cholesterol measurements, without a fasting specimen, makes it particularly suitable as a monitoring target for statin therapy in primary prevention settings.

For guideline developers, this study emphasizes that the residual risk of ASCVD among statin-treated patients in primary prevention could be further reduced by achieving contemporary guideline-recommended goals of at least an LDL cholesterol <2.6 mmol/L or a non-HDL cholesterol <3.4 mmol/L [25,26]. Although guideline-endorsed targets were used to contextualize where on the continuous dose-response curve the relevant policy decision falls, our continuous analysis showed that the dose-response relationship for MACE was approximately log-linear across the full spectrum of achieved LDL and non-HDL cholesterol. While guideline recommendations have evolved since our cohort enrolment period (2007–2017), our findings do imply that if these atherogenic cholesterol concentrations are selected as a minimal target to evaluate statin effectiveness in primary prevention, then nearly 40% of patients will require additional therapy with a non-statin lipid-lowering medication, such as ezetimibe or PCSK9 inhibitors, or intensification of dietary and other lifestyle interventions, to achieve their treatment goal. The optimal individual cholesterol target will, however, depend on clinical judgement, patient comorbidities, medication tolerability, and local cost-effectiveness considerations.

There are limitations to our study. Although we pooled data from three databases, our estimates at the lowest concentrations of atherogenic cholesterol were imprecise because of the low number of patients exposed after starting statins. As with any observational study, residual confounding is possible. Clinicians intensify therapy according to a patient’s total cardiovascular risk. The lowest achieved concentrations will be enriched for higher-risk patients (confounding by indication), and medical conditions that lower cholesterol such as malignancies, frailty, liver disease or heart failure, may also increase the risk of subsequent cardiovascular events and all-cause mortality (reverse causation) [33]. Both mechanisms could bias estimates at the lowest concentrations towards harm. Although the new-user and landmark design used in our study mitigates these biases, we could not account for all comorbidities. Few patients achieved an LDL cholesterol <1.4 mmol/L at the landmark, and these individuals were older and had more comorbidities corresponding to their treatment with more intensive statin therapy. Given the small absolute numbers of events at the extremely low concentrations of atherogenic cholesterol, these estimates should not be over-interpreted. We acknowledge that electronic health record and administrative data do not readily capture lifestyle changes or whether comorbidities worsened between statin initiation and the landmark, which may have contributed to the association with MACE and all-cause mortality at extremely low concentrations of atherogenic cholesterol. Although we controlled for diagnosis of chronic liver disease, we did not control for malignancy or low BMI (HK and NZ cohorts), which could both contribute to wasting and confound the relationship between very-low LDL cholesterol and all-cause mortality. However, our sensitivity analysis using the UK cohort was robust to further adjustment of several clinically relevant variables including BMI. We also could not compare ethnic groups across databases because of varied definitions and missing data, but our findings suggest limited differences between HK, which is predominantly Han Chinese, and the UK, which is predominantly White. The choice of a two-year landmark may have contributed to selection bias notably for patients with very low atherogenic cholesterol, who also have the worst prognosis because they may be selectively excluded (if they experience MACE or die early) or selectively retained (if they survive to the landmark despite illness).

We observed clinical and statistical heterogeneity across cohorts, most notably at the lowest concentrations of achieved cholesterol levels. All three data sources shared limitations inherent to routinely collected health data. Inclusion in the study required a follow-up lipid test, which excluded otherwise eligible patients in all three cohorts, and patients who had a follow-up lipid test tend to be more adherent to therapy. Each cohort therefore represented a defined rather than a fully representative population: public-sector care in HK, contributing practices in CPRD, and laboratory data in NZ from the Auckland and Northland regions, with coverage gaps in follow-up lipid testing [34,35]. Such selection in cohorts could influence absolute risk and generalisability across all categories of achieved atherogenic cholesterol. The cohorts differ in two aspects that directly impact on the association between achieved cholesterol level and outcomes. Firstly, lipid management practices differed between the three regions. Throughout the study period, NZ guidance recommended treatment intensity according to absolute 5-year cardiovascular risk, with LDL cholesterol goals of <2.5 mmol/L (2003) and <2.0 mmol/L (2009) and very-high-risk primary prevention patients were managed as for established CVD [36,37]. In Auckland and Northland regions, this approach was supported by the PREDICT decision-support system from 2002 [38]. By contrast, UK guidance recommended fixed-dose statin therapy without a cholesterol target in primary prevention, while in HK risk-stratified, target-based management was introduced mainly for patients with diabetes [39,40]. Where treatment intensity is guided by absolute CVD risk, patients achieving the lowest cholesterol levels are more likely to have the highest underlying risk, biasing associations at low concentrations towards harm due to confounding by indication. Additionally, diagnosis in NZ were available only from public hospital records and dispensing data, so comorbidities and cardiovascular diseases managed in primary care were less completely recorded than in HK and the UK, increasing the potential for residual confounding and for misclassification of some higher-risk patients as primary prevention. Cardiovascular risk estimates from PREDICT were not available for this study hence we could not adjust for the risk assessment that guided treatment in NZ.

In the NZ cohort, the crude rate of MACE was approximately 2-fold higher in the <1.4 mmol/L than in the ≥2.6 mmol/L LDL cholesterol category (18.45 versus 9.16 per 1,000 person-years), a gradient not seen in HK or the UK cohorts and is larger than would be expected from LDL cholesterol alone. In the NZ cohort, the higher risk of MACE at LDL cholesterol <1.4 mmol/L was confined to patients initiated on moderate- or high-intensity statins and was not observed among those initiated on low-intensity statins or among patients with diabetes, whereas the point estimates were below one in each of these subgroups. The associations at low concentrations in the NZ cohort therefore may reflect confounding by indication, together with reverse causation and residual confounding. Excluding the NZ cohort shifted estimates at the lowest concentrations towards greater benefit and reduced heterogeneity. These findings show that associations between achieved cholesterol and outcomes in observational data depend on how treatment intensity is allocated within each health system, and underscore the value of including diverse populations and data sources to assess the consistency of such associations [41]. Lastly, our findings should not be extrapolated to statin treatment-naïve populations, to populations who initiate statins but do not have a measurable lipid response during follow-up, or to populations with different healthcare access characteristics. We also encourage others to replicate our findings in other databases containing high-quality longitudinal data on medication use, laboratory tests, and cardiovascular and mortality outcomes.

In conclusion, among statin-treated patients in primary prevention, the absolute concentration of achieved atherogenic cholesterol was consistently associated with a reduction in MACE without an increased risk of all-cause mortality. After initiating statins for primary prevention, achieving guideline-recommended targets of LDL or non-HDL cholesterol may be associated with reductions in population-level morbidity and mortality from ASCVD.

## Supporting information

S1 Checklist Reporting of Studies Conducted using Observational Routinely Collected Data (RECORD) guideline checklist. (DOCX)

S1 Text Supplementary tables and figures. (DOCX)

S1 Code Data analysis code. (ZIP)

## Abbreviations

BMI: body mass index
CI: confidence intervals
CPRD: Clinical Practice Research Datalink
HDL: high-density lipoprotein
HR: hazard ratios
LDL: low-density lipoprotein
MACE: major adverse cardiovascular events
RCTs: randomized controlled trials
SD: standard deviation

## References

[1] Vaduganathan M, Mensah GA, Turco JV, Fuster V, Roth GA. The global burden of cardiovascular diseases and risk. J Am Coll Cardiol. 2022;80(25):2361–71. doi: 10.1016/j.jacc.2022.11.005 36368511

[2] Ference BA, Ginsberg HN, Graham I, Ray KK, Packard CJ, Bruckert E, et al. Low-density lipoproteins cause atherosclerotic cardiovascular disease. 1. Evidence from genetic, epidemiologic, and clinical studies. A consensus statement from the European Atherosclerosis Society Consensus Panel. Eur Heart J. 2017;38(32):2459–72. doi: 10.1093/eurheartj/ehx144 28444290 PMC5837225

[3] Brunner FJ, Waldeyer C, Ojeda F, Salomaa V, Kee F, Sans S, et al. Application of non-HDL cholesterol for population-based cardiovascular risk stratification: results from the Multinational Cardiovascular Risk Consortium. Lancet. 2019;394(10215):2173–83. doi: 10.1016/S0140-6736(19)32519-X 31810609 PMC6913519

[4] Stone NJ, Robinson JG, Lichtenstein AH, Bairey Merz CN, Blum CB, Eckel RH, et al. 2013 ACC/AHA guideline on the treatment of blood cholesterol to reduce atherosclerotic cardiovascular risk in adults: A report of the American College of Cardiology/American Heart Association Task Force on Practice Guidelines. J Am Coll Cardiol. 2014;63(25 Pt B):2889–934. doi: 10.1016/j.jacc.2013.11.002 24239923

[5] Waters DD. Cholesterol lowering guidelines: From whence we came and where we are now. Can J Cardiol. 2019;35(5):590–7. doi: 10.1016/j.cjca.2018.07.477 30454967

[6] Kolber MR, Klarenbach S, Cauchon M, Cotterill M, Regier L, Marceau RD, et al. PEER simplified lipid guideline 2023 update: Prevention and management of cardiovascular disease in primary care. Can Fam Physician. 2023;69(10):675–86. doi: 10.46747/cfp.6910675 37833089 PMC10575658

[7] Boekholdt SM, Hovingh GK, Mora S, Arsenault BJ, Amarenco P, Pedersen TR, et al. Very low levels of atherogenic lipoproteins and the risk for cardiovascular events: a meta-analysis of statin trials. J Am Coll Cardiol. 2014;64(5):485–94. doi: 10.1016/j.jacc.2014.02.615 25082583 PMC4443441

[8] Boekholdt SM, Arsenault BJ, Mora S, Pedersen TR, LaRosa JC, Nestel PJ, et al. Association of LDL cholesterol, non-HDL cholesterol, and apolipoprotein B levels with risk of cardiovascular events among patients treated with statins: A meta-analysis. JAMA. 2012;307(12):1302–9. doi: 10.1001/jama.2012.366 22453571

[9] Matsuzaki M, Kita T, Mabuchi H, Matsuzawa Y, Nakaya N, Oikawa S, et al. Large scale cohort study of the relationship between serum cholesterol concentration and coronary events with low-dose simvastatin therapy in Japanese patients with hypercholesterolemia. Circ J. 2002;66(12):1087–95. doi: 10.1253/circj.66.1087 12499611

[10] Johannesen CDL, Langsted A, Mortensen MB, Nordestgaard BG. Association between low density lipoprotein and all cause and cause specific mortality in Denmark: Prospective cohort study. BMJ. 2020;371:m4266. doi: 10.1136/bmj.m4266 33293274 PMC7722479

[11] Johannesen CDL, Mortensen MB, Langsted A, Nordestgaard BG. Apolipoprotein B and non-HDL cholesterol better reflect residual risk than LDL cholesterol in statin-treated patients. J Am Coll Cardiol. 2021;77(11):1439–50. doi: 10.1016/j.jacc.2021.01.027 33736827

[12] Spitz J, Patel J, Agarwala A, Sharma G, Mehta A, Natarajan P, et al. A critical appraisal of lipid management in the post-statin era: Comparison on guidelines, therapeutic targets, and screening in a case-based framework of lipid management. JACC Adv. 2025;4(6 Pt 2):101823. doi: 10.1016/j.jacadv.2025.101823 40579058 PMC12277597

[13] Blais JE, Akyea RK, Coetzee A, Chan AH, Lau WC, Man KK, et al. Lipid levels and major adverse cardiovascular events in patients initiated on statins for primary prevention: An international population-based cohort study protocol. BJGP Open. 2021;5(1):bjgpopen20X101127. doi: 10.3399/bjgpopen20X101127 33172851 PMC7960514

[14] Lai EC-C, Man KKC, Chaiyakunapruk N, Cheng C-L, Chien H-C, Chui CSL, et al. Brief report: Databases in the Asia-Pacific Region: The potential for a distributed network approach. Epidemiology. 2015;26(6):815–20. doi: 10.1097/EDE.0000000000000325 26133022

[15] Herrett E, Gallagher AM, Bhaskaran K, Forbes H, Mathur R, van Staa T. Data resource profile: Clinical practice research datalink (CPRD). Int J Epidemiol. 2015;44(3):827–36. doi: 10.1093/ije/dyv098 PMC4521131 26050254

[16] Sanchez-Santos MT, Axson EL, Dedman D, Delmestri A. Data resource profile update: CPRD GOLD. Int J Epidemiol. 2025;54(4). doi: 10.1093/ije/dyaf077 PMC12158158 40499193

[17] Dafni U. Landmark analysis at the 25-year landmark point. Circ Cardiovasc Qual Outcomes. 2011;4(3):363–71. doi: 10.1161/CIRCOUTCOMES.110.957951 21586725

[18] Akyea RK, Kai J, Qureshi N, Iyen B, Weng SF. Sub-optimal cholesterol response to initiation of statins and future risk of cardiovascular disease. Heart. 2019;105(13):975–81. doi: 10.1136/heartjnl-2018-314253 30988003 PMC6582718

[19] Yourman LC, Cenzer IS, Boscardin WJ, Nguyen BT, Smith AK, Schonberg MA, et al. Evaluation of time to benefit of statins for the primary prevention of cardiovascular events in adults aged 50 to 75 years: A meta-analysis. JAMA Intern Med. 2021;181(2):179–85. doi: 10.1001/jamainternmed.2020.6084 33196766 PMC7670393

[20] Jacobson TA, Ito MK, Maki KC, Orringer CE, Bays HE, Jones PH, et al. National lipid association recommendations for patient-centered management of dyslipidemia: Part 1--full report. J Clin Lipidol. 2015;9(2):129–69. doi: 10.1016/j.jacl.2015.02.003 25911072

[21] Mach F, Baigent C, Catapano AL, Koskinas KC, Casula M, Badimon L, et al. 2019 ESC/EAS Guidelines for the management of dyslipidaemias: Lipid modification to reduce cardiovascular risk. Eur Heart J. 2020;41(1):111–88. doi: 10.1093/eurheartj/ehz455 31504418

[22] Grundy SM, Stone NJ, Bailey AL, Beam C, Birtcher KK, Blumenthal RS, et al. 2018 AHA/ACC/AACVPR/AAPA/ABC/ACPM/ADA/AGS/APhA/ASPC/NLA/PCNA guideline on the management of blood cholesterol: A report of the American College of Cardiology/American Heart Association Task Force on Clinical Practice Guidelines. Circulation. 2019;139(25):e1082–143. doi: 10.1161/CIR.0000000000000625 PMC7403606 30586774

[23] Viechtbauer W. Conducting meta-analyses in R with the metafor package. J Stat Softw. 2010;1(3).

[24] Giugliano RP, Pedersen TR, Park J-G, De Ferrari GM, Gaciong ZA, Ceska R, et al. Clinical efficacy and safety of achieving very low LDL-cholesterol concentrations with the PCSK9 inhibitor evolocumab: A prespecified secondary analysis of the FOURIER trial. Lancet. 2017;390(10106):1962–71. doi: 10.1016/S0140-6736(17)32290-0 28859947

[25] Blumenthal RS, Morris PB, Gaudino M, Johnson HM, Anderson TS, Bittner VA, et al. 2026 ACC/AHA/AACVPR/ABC/ACPM/ADA/AGS/APhA/ASPC/NLA/PCNA guideline on the management of dyslipidemia: A report of the American College of Cardiology/American Heart Association Joint Committee on Clinical Practice Guidelines. Circulation. 2026;153(17):e1154–276. doi: 10.1161/CIR.0000000000001423 41824552

[26] Mach F, Koskinas KC, Roeters van Lennep JE, Tokgözoğlu L, Badimon L, Baigent C, et al. 2025 Focused Update of the 2019 ESC/EAS Guidelines for the management of dyslipidaemias. Eur Heart J. 2025;46(42):4359–78. doi: 10.1093/eurheartj/ehaf190 40878289

[27] Rana JS, Liu JY, Moffet HH, Boklage SH, Khan I, Karter AJ. Risk of incident atherosclerotic cardiovascular disease events by achieved atherogenic lipid levels among 62,428 statin-treated individuals with diabetes mellitus. Am J Cardiol. 2018;122(5):762–7. doi: 10.1016/j.amjcard.2018.05.011 30057224

[28] Kim MK, Han K, Joung HN, Baek KH, Song KH, Kwon HS. Cholesterol levels and development of cardiovascular disease in Koreans with type 2 diabetes mellitus and without pre-existing cardiovascular disease. Cardiovasc Diabetol. 2019;18(1):139. doi: 10.1186/s12933-019-0943-9 PMC6805335 31640795

[29] Somani S, van Buchem MM, Sarraju A, Hernandez-Boussard T, Rodriguez F. Artificial intelligence-enabled analysis of statin-related topics and sentiments on social media. JAMA Netw Open. 2023;6(4):e239747. doi: 10.1001/jamanetworkopen.2023.9747 PMC10126874 37093597

[30] Sniderman AD, St-Pierre AC, Cantin B, Dagenais GR, Després J-P, Lamarche B. Concordance/discordance between plasma apolipoprotein B levels and the cholesterol indexes of atherosclerotic risk. Am J Cardiol. 2003;91(10):1173–7. doi: 10.1016/s0002-9149(03)00262-5 12745098

[31] Sniderman AD, Williams K, Contois JH, Monroe HM, McQueen MJ, de Graaf J, et al. A meta-analysis of low-density lipoprotein cholesterol, non-high-density lipoprotein cholesterol, and apolipoprotein B as markers of cardiovascular risk. Circ Cardiovasc Qual Outcomes. 2011;4(3):337–45. doi: 10.1161/CIRCOUTCOMES.110.959247 21487090

[32] Langlois MR, Chapman MJ, Cobbaert C, Mora S, Remaley AT, Ros E, et al. Quantifying atherogenic lipoproteins: Current and future challenges in the era of personalized medicine and very low concentrations of LDL cholesterol. A consensus statement from EAS and EFLM. Clin Chem. 2018;64(7):1006–33. doi: 10.1373/clinchem.2018.287037 29760220

[33] Jacobs D, Blackburn H, Higgins M, Reed D, Iso H, McMillan G, et al. Report of the conference on low blood cholesterol: Mortality associations. Circulation. 1992;86(3):1046–60. doi: 10.1161/01.CIR.86.3.1046 1355411

[34] Exeter DJ, Moss L, Zhao J, Kyle C, Riddell T, Jackson R, et al. The distribution and frequency of blood lipid testing by sociodemographic status among adults in Auckland, New Zealand. J Prim Health Care. 2015;7(3):182–91. doi: 10.1071/hc15182 26437041

[35] Beadel H, Halim A, Bridgford P, Hudson B. Lipid monitoring in a community cohort of people taking statins: who is tested and is testing associated with subsequent alteration in therapy?. N Z Med J. 2020;133(1518):54–63. 32683432

[36] National Heart Foundation of New Zealand, Stroke Foundation of New Zealand, New Zealand. Ministry of Health, New Zealand Guidelines Group. The assessment and management of cardiovascular risk. Wellington, N.Z.: New Zealand Guidelines Group; 2003.

[37] New Zealand Guidelines Group; New Zealand Ministry of Health. New Zealand cardiovascular guidelines handbook: A summary resource for primary care practitioners. Wellington, N.Z.: New Zealand Guidelines Group; 2009.

[38] Wells S, Riddell T, Kerr A, Pylypchuk R, Chelimo C, Marshall R, et al. Cohort profile: The PREDICT cardiovascular disease cohort in New Zealand primary care (PREDICT-CVD 19). Int J Epidemiol. 2017;46(1):22. doi: 10.1093/ije/dyv312 26686841

[39] Cooper A, O’Flynn N, Guideline Development Group. Risk assessment and lipid modification for primary and secondary prevention of cardiovascular disease: Summary of NICE guidance. BMJ. 2008;336(7655):1246–8. doi: 10.1136/bmj.39554.624086.AD 18511800 PMC2405875

[40] Wong MCS, Sin CKL, Lee JPM. The reference framework for diabetes care in primary care settings. Hong Kong Med J. 2012;18(3):238–46. 22665689

[41] Madigan D, Ryan PB, Schuemie M, Stang PE, Overhage JM, Hartzema AG, et al. Evaluating the impact of database heterogeneity on observational study results. Am J Epidemiol. 2013;178(4):645–51. doi: 10.1093/aje/kwt010 23648805 PMC3736754

