# Supplementary material for: Risk of cardiovascular events and mortality associated with LDL and non-HDL cholesterol levels after statin initiation for primary prevention: A multiregional cohort study
Source: PLoS Med. 2026 Nov 3;23(11):e1005012. doi: 10.1371/journal.pmed.1005012 (PMC13766616; doi:10.1371/journal.pmed.1005012)
Supplement: Supplementary file 2 [file pmed.1005012.s002.docx]

# S1 Text

**Table A:** Baseline characteristics for patients in each cohort according to achieved low-density lipoprotein cholesterol at the year 2 landmark.

|  | **Hong Kong (n=101411)** | | | | **United Kingdom (n=45907)** | | | | **New Zealand (n=37894)** | | | |
| --- | --- | --- | --- | --- | --- | --- | --- | --- | --- | --- | --- | --- |
|  | Year 2 LDL-C, mmol/L | | | | Year 2 LDL-C, mmol/L | | | | Year 2 LDL-C, mmol/L | | | |
| **Characteristic** | <1.4 | 1.4-1.7 | 1.8-2.5 | ≥2.6 | <1.4 | 1.4-1.7 | 1.8-2.5 | ≥2.6 | <1.4 | 1.4-1.7 | 1.8-2.5 | ≥2.6 |
| Number of patients | 2731 (2.7%) | 11911 (11.7%) | 48768 (48.1%) | 38001 (37.5%) | 2940 (6.4%) | 6092 (13.3%) | 18113 (39.5%) | 18762 (40.9%) | 1413 (3.7%) | 3890 (10.3%) | 15060 (39.7%) | 17531 (46.3%) |
| Sex |  |  |  |  |  |  |  | - |  |  |  |  |
| Men | 1269 (46.5%) | 5616 (47.1%) | 23085 (47.3%) | 17154 (45.1%) | 1978 (67.3%) | 3786 (62.1%) | 10455 (57.7%) | 10271 (54.7%) | 895 (63.3%) | 2212 (56.9%) | 7950 (52.8%) | 9120 (52.0%) |
| Women | 1462 (53.5%) | 6295 (52.9%) | 25683 (52.7%) | 20847 (54.9%) | 962 (32.7%) | 2306 (37.9%) | 7658 (42.3%) | 8491 (45.3%) | 518 (36.7%) | 1678 (43.1%) | 7110 (47.2%) | 8411 (48.0%) |
| Ethnicity |  |  |  |  |  |  |  |  |  |  |  |  |
| Chinese (HK) | 2519 (92.2%) | 10918 (91.7%) | 44546 (91.3%) | 34754 (91.5%) | - | - | - | - | - |  | - | - |
| Other ethnicity (HK) | 212 (7.8%) | 993 (8.3%) | 4222 (8.7%) | 3247 (8.5%) | - | - | - | - | - |  | - | - |
| Māori (NZ) | - | - | - | - | - | - |  | - | 113 (8.0%) | 257 (6.6%) | 959 (6.4%) | 1228 (7.0%) |
| Pacific (NZ) | - | - | - | - | - | - | - | - | 206 (14.6%) | 482 (12.4%) | 1485 (9.9%) | 1791 (10.2%) |
| All Asian (NZ) | - | - | - | - | - | - | - | - | 342 (24.2%) | 1072 (27.6%) | 3339 (22.2%) | 3342 (19.1%) |
| European and other (NZ) | - | - | - | - | - | - | - | - | 752 (53.2%) | 2079 (53.4%) | 9277 (61.6%) | 11170 (63.7%) |
| Asian/Asian British/other Asian (UK) | - | - | - | - | 46 (1.6%) | 70 (1.2%) | 216 (1.2%) | 216 (1.2%) | - | - | - | - |
| Black/African/Caribbean (UK) | - | - | - | - | 28 (1.0%) | 40 (0.7%) | 110 (0.6%) | 166 (0.9%) | - | - | - | - |
| Mixed/multiple (UK) | - | - | - | - | 68 (2.3%) | 86 (1.4%) | 228 (1.3%) | 206 (1.1%) | - | - | - | - |
| Other ethnic group (UK) | - | - | - | - | 23 (0.8%) | 47 (0.8%) | 118 (0.7%) | 138 (0.7%) | - | - | - | - |
| White (UK) | - | - | - | - | 719 (24.5%) | 1649 (27.1%) | 4961 (27.4%) | 5200 (27.7%) | - | - | - | - |
| Unknown (UK) | - | - | - | - | 2056 (69.9%) | 4200 (68.9%) | 12480 (68.9%) | 12836 (68.4%) | - | - | - | - |
| Age, years (mean (SD)) | 64.7 (11.3) | 64.2 (10.9) | 62.7 (10.8) | 60.6 (11.1) | 60.8 (11.9) | 61.1 (11.4) | 60.1 (10.9) | 57.5 (11.1) | 61.0 (12.4) | 60.2 (11.6) | 58.8 (11.3) | 56.0 (11.2) |
| Body mass index (mean (SD))* | - | - | - | - | 30 (6.0) | 29.7 (6.1) | 29.2 (5.7) | 29 (5.4) | - | - | - | - |
| Systolic blood pressure (mean (SD))* | - | - | - | - | 140 (19.0) | 140 (18.0) | 140 (18.0) | 139 (18.0) | - | - | - | - |
| Diastolic blood pressure (mean (SD))* | - | - | - | - | 82 (11.0) | 82 (10.0) | 82 (11.0) | 83 (10.0) | - | - | - | - |
| **Lipid tests at time of statin initiation** |  |  |  |  |  |  |  |  |  |  |  |  |
| Total cholesterol, mmol/L (mean (SD)) | 5.2 (1.0) | 5.4 (0.9) | 5.7 (0.9) | 6.4 (1.1) | 5.1 (1.2) | 5.7 (1.1) | 6.3 (1.2) | 6.9 (1.3) | 4.8 (1.1) | 5.3 (1.0) | 5.8 (1.0) | 6.5 (1.0) |
| LDL-C, mmol/L (mean (SD)) | 3.1 (0.8) | 3.3 (0.7) | 3.7 (0.8) | 4.3 (1.0) | 2.9 (1.0) | 3.5 (1.0) | 4.1 (1.5) | 4.7 (1.1) | 2.7 (0.8) | 3.2 (0.8) | 3.7 (0.8) | 4.3 (0.9) |
| LDL-C group, mmol/L (number (%)) |  |  |  |  |  |  |  |  |  |  |  |  |
| <1.4 | 100 (3.7%) | 112 (0.9%) | 103 (0.2%) | 26 (0.1%) | 250 (8.5%) | 118 (1.9%) | 72 (0.4%) | 29 (0.2%) | 79 (5.6%) | 63 (1.6%) | 42 (0.3%) | 14 (0.1%) |
| 1.4-1.7 | 116 (4.2%) | 249 (2.1%) | 441 (0.9%) | 103 (0.3%) | 217 (7.4%) | 291 (4.8%) | 278 (1.5%) | 56 (0.3%) | 91 (6.4%) | 132 (3.4%) | 187 (1.2%) | 54 (0.3%) |
| 1.8-2.5 | 255 (9.3%) | 650 (5.5%) | 2141 (4.4%) | 999 (2.6%) | 617 (21%) | 701 (11.5%) | 1382 (7.6%) | 590 (3.1%) | 451 (31.9%) | 659 (16.9%) | 1087 (7.2%) | 497 (2.8%) |
| ≥2.6 | 2260 (82.8%) | 10900 (91.5%) | 46083 (94.5%) | 36873 (97%) | 1856 (63.1%) | 4982 (81.8%) | 16381 (90.4%) | 18087 (96.4%) | 792 (56.1%) | 3036 (78.1%) | 13744 (91.3%) | 16966 (96.8%) |
| non-HDL-C, mmol/L (mean (SD)) | 3.9 (1.0) | 4.1 (0.8) | 4.4 (0.9) | 5.0 (1.1) | 3.8 (1.2) | 4.3 (1.1) | 4.9 (1.2) | 5.5 (1.3) | 3.6 (1.0) | 4.0 (0.9) | 4.5 (0.9) | 5.1 (1.0) |
| non-HDL-C group, mmol/L (number (%)) |  |  |  |  |  |  |  |  |  |  |  |  |
| <2.2 | 123 (4.5%) | 232 (1.9%) | 330 (0.7%) | 67 (0.2%) | 294 (10.0%) | 226 (3.7%) | 234 (1.3%) | 110 (0.6%) | 107 (7.6%) | 141 (3.6%) | 132 (0.9%) | 45 (0.3%) |
| 2.2-2.5 | 88 (3.2%) | 230 (1.9%) | 636 (1.3%) | 214 (0.6%) | 155 (5.3%) | 293 (4.8%) | 425 (2.4%) | 109 (0.6%) | 96 (6.8%) | 155 (4.0%) | 263 (1.8%) | 86 (0.5%) |
| 2.6-3.3 | 388 (14.2%) | 1359 (11.4%) | 3377 (6.9%) | 1458 (3.8%) | 490 (16.7%) | 596 (9.8%) | 1174 (6.5%) | 639 (3.4%) | 389 (27.5%) | 677 (17.4%) | 1220 (8.1%) | 602 (3.4%) |
| ≥3.4 | 2132 (78.1%) | 10090 (84.7%) | 44425 (91.1%) | 36262 (95.4%) | 2001 (68.1%) | 4977 (81.7%) | 16280 (89.9%) | 17904 (95.4%) | 821 (58.1%) | 2917 (75.0%) | 13445 (89.3%) | 16798 (95.8%) |
| HDL-C, mmol/L (mean (SD)) | 1.3 (0.4) | 1.3 (0.4) | 1.3 (0.3) | 1.3 (0.3) | 1.3 (0.5) | 1.3 (0.5) | 1.4 (0.5) | 1.4 (0.5) | 1.2 (0.4) | 1.3 (0.4) | 1.4 (0.4) | 1.4 (0.4) |
| Triglycerides, mmol/L (median (IQR)) | 1.6 (1.2) | 1.4 (0.9) | 1.4 (0.9) | 1.5 (1.0) | 2.1 (1.3) | 2.0 (1.0) | 1.9 (1.4) | 2.0 (1.2) | 1.9 (1.0) | 1.7 (0.9) | 1.7 (0.8) | 1.8 (0.8) |
| **Comorbidities** |  |  |  |  |  |  |  |  |  |  |  |  |
| Hypertension | 1669 (61.1%) | 6816 (57.2%) | 25387 (52.1%) | 17240 (45.4%) | 918 (31.2%) | 1791 (29.4%) | 5095 (28.1%) | 4526 (24.1%) | 633 (44.8%) | 1721 (44.2%) | 5636 (37.4%) | 4977 (28.4%) |
| Atrial fibrillation | 37 (1.4%) | 169 (1.4%) | 718 (1.5%) | 498 (1.3%) | 86 (2.9%) | 144 (2.4%) | 407 (2.3%) | 340 (1.8%) | 60 (4.3%) | 88 (2.3%) | 302 (2.0%) | 231 (1.3%) |
| Diabetes mellitus | 2182 (79.9%) | 9059 (76.1%) | 30758 (63.1%) | 16137 (42.5%) | 1416 (48.2%) | 2077 (34.1%) | 3632 (20.1%) | 2448 (13.1%) | 326 (23.1%) | 750 (19.3%) | 1420 (9.4%) | 937 (5.3%) |
| Mental health disorder | 263 (9.6%) | 918 (7.7%) | 3540 (7.3%) | 2985 (7.9%) | 43 (1.5%) | 65 (1.1%) | 193 (1.1%) | 213 (1.1%) | 97 (6.9%) | 247 (6.4%) | 1051 (7%) | 1415 (8.1%) |
| Obesity | 35 (1.3%) | 109 (0.9%) | 409 (0.8%) | 347 (0.9%) | 211 (7.2%) | 383 (6.3%) | 950 (5.2%) | 1010 (5.4%) | 38 (2.7%) | 112 (2.9%) | 252 (1.7%) | 310 (1.8%) |
| Dyslipidemia | 105 (3.8%) | 456 (3.8%) | 2386 (4.9%) | 2642 (7.0%) | 344 (11.7%) | 1036 (17.0%) | 4277 (23.6%) | 5787 (30.8%) | 22 (1.6%) | 86 (2.2%) | 297 (2%) | 427 (2.4%) |
| Smoking | 10 (0.4%) | 70 (0.6%) | 281 (0.6%) | 212 (0.6%) | 893 (30.4%) | 1819 (29.9%) | 5398 (29.8%) | 6228 (33.1%) | 16 (1.1%) | 32 (0.8%) | 143 (1.0%) | 138 (0.8%) |
| COPD# | 42 (1.5%) | 179 (1.5%) | 627 (1.3%) | 583 (1.5%) | - | - | - | - | 32 (2.3%) | 76 (2.0%) | 223 (1.5%) | 193 (1.1%) |
| Chronic liver disease | 79 (2.9%) | 269 (2.3%) | 1154 (2.4%) | 867 (2.3%) | 34 (1.2%) | 78 (1.3%) | 189 (1.0%) | 182 (1.0%) | 4 (0.3%) | 8 (0.2%) | 24 (0.2%) | 29 (0.2%) |
| Chronic pancreatitis | 2 (0.1%) | 6 (0.1%) | 13 (0.0%) | 13 (0.0%) | 13 (0.4%) | 28 (0.5%) | 63 (0.4%) | 47 (0.3%) | 2 (0.1%) | 7 (0.2%) | 7 (0.1%) | 10 (0.1%) |
| Rheumatoid arthritis and related disorders | 3 (0.1%) | 27 (0.2%) | 175 (0.4%) | 275 (0.7%) | 26 (0.9%) | 51 (0.8%) | 145 (0.8%) | 135 (0.7%) | 2 (0.1%) | 4 (0.1%) | 15 (0.1%) | 24 (0.1%) |
| SLE | 8 (0.3%) | 21 (0.2%) | 126 (0.3%) | 161 (0.4%) | 4 (0.1%) | 6 (0.1%) | 18 (0.1%) | 24 (0.1%) | 4 (0.3%) | 6 (0.2%) | 45 (0.3%) | 29 (0.2%) |
| Alcohol misuse | 22 (0.8%) | 0 (0.0%) | 215 (0.4%) | 193 (0.5%) | 75 (2.6%) | 131 (2.2%) | 387 (2.1%) | 476 (2.5%) | 26 (1.8%) | 40 (1.0%) | 173 (1.2%) | 156 (0.9%) |
| History of substance use# | 30 (1.1%) | 82 (0.7%) | 307 (0.6%) | 269 (0.7%) | - | - | - | - | 17 (1.2%) | 13 (0.3%) | 78 (0.5%) | 72 (0.4%) |
| **Medications** |  |  |  |  |  |  |  |  |  |  |  |  |
| Statin drug |  |  |  |  |  |  |  |  |  |  |  |  |
| Atorvastatin | 92 (3.4%) | 278 (2.3%) | 1228 (2.5%) | 1390 (3.7%) | 173 (5.9%) | 376 (6.2%) | 989 (5.5%) | 994 (5.3%) | 490 (34.7%) | 1232 (31.7%) | 3609 (24%) | 3385 (19.3%) |
| Fluvastatin | 3 (0.1%) | 1 (0.0%) | 12 (0.0%) | 19 (0.0%) | 6 (0.2%) | 17 (0.3%) | 76 (0.4%) | 121 (0.6%) | 0 (0.0%) | 0 (0.0%) | 0 (0.0%) | 0 (0.0%) |
| Lovastatin | 0 (0.0%) | 0 (0.0%) | 1 (0.0%) | 0 (0.0%) | 0 (0.0%) | 0 (0.0%) | 0 (0.0%) | 0 (0.0%) | 0 (0.0%) | 0 (0.0%) | 0 (0.0%) | 0 (0.0%) |
| Pravastatin | 0 (0.0%) | 0 (0.0%) | 1 (0.0%) | 2 (0.0%) | 52 (1.8%) | 104 (1.7%) | 300 (1.7%) | 459 (2.5%) | 1 (0.1%) | 1 (0.0%) | 21 (0.1%) | 70 (0.4%) |
| Rosuvastatin | 78 (2.9%) | 169 (1.4%) | 428 (0.9%) | 449 (1.2%) | 786 (26.7%) | 1645 (27%) | 4971 (27.4%) | 5028 (26.8%) | 0 (0.0%) | 0 (0.0%) | 0 (0.0%) | 0 (0.0%) |
| Simvastatin | 2558 (93.7%) | 11463 (96.2%) | 47098 (96.6%) | 36141 (95.1%) | 1923 (65.4%) | 3950 (64.8%) | 11777 (65%) | 12160 (64.8%) | 922 (65.3%) | 2657 (68.3%) | 11430 (75.9%) | 14076 (80.3%) |
| Statin intensity |  |  |  |  |  |  |  |  |  |  |  |  |
| Low | 2094 (76.7%) | 9708 (81.5%) | 40347 (82.7%) | 29885 (78.6%) | 111 (3.8%) | 255 (4.2%) | 1196 (6.6%) | 1577 (8.4%) | 261 (18.5%) | 751 (19.3%) | 3413 (22.7%) | 4510 (25.7%) |
| Moderate | 625 (22.9%) | 2180 (18.3%) | 8371 (17.2%) | 8038 (21.2%) | 2678 (91.1%) | 5505 (90.4%) | 16131 (89.1%) | 16409 (87.5%) | 1000 (70.8%) | 2869 (73.8%) | 11154 (74.1%) | 12635 (72.1%) |
| High | 12 (0.4%) | 23 (0.2%) | 50 (0.1%) | 78 (0.2%) | 151 (5.1%) | 332 (5.5%) | 786 (4.3%) | 776 (4.1%) | 152 (10.8%) | 270 (6.9%) | 493 (3.3%) | 386 (2.2%) |
| Other lipid-lowering drugs | 4 (0.1%) | 4 (0.0%) | 14 (0.0%) | 23 (0.1%) | 11 (0.4%) | 19 (0.3%) | 38 (0.2%) | 49 (0.3%) | 28 (2.0%) | 70 (1.8%) | 231 (1.5%) | 435 (2.5%) |
| Diabetes drug | 2149 (78.7%) | 8917 (74.9%) | 30205 (61.9%) | 15714 (41.4%) | 1244 (42.3%) | 1745 (28.6%) | 2979 (16.5%) | 1928 (10.3%) | 269 (19.0%) | 627 (16.1%) | 1133 (7.5%) | 710 (4.1%) |
| Insulin# | 243 (8.9%) | 846 (7.1%) | 2579 (5.3%) | 1646 (4.3%) | - | - | - | - | 39 (2.8%) | 87 (2.2%) | 209 (1.4%) | 156 (0.9%) |
| Non-insulin diabetes drug# | 2122 (77.7%) | 8817 (74%) | 29855 (61.2%) | 15425 (40.6%) | - | - | - | - | 254 (18%) | 581 (14.9%) | 988 (6.6%) | 597 (3.4%) |
| Aspirin# | 471 (17.2%) | 1619 (13.6%) | 5568 (11.4%) | 3722 (9.8%) | - | - | - | - | 248 (17.6%) | 604 (15.5%) | 1693 (11.2%) | 1178 (6.7%) |
| Other antiplatelet drugs (All antiplatelet drugs in UK) | 32 (1.2%) | 74 (0.6%) | 199 (0.4%) | 126 (0.3%) | 916 (31.2%) | 1562 (25.6%) | 3393 (18.7%) | 2370 (12.6%) | 6 (0.4%) | 21 (0.5%) | 25 (0.2%) | 17 (0.1%) |
| Oral anticoagulants | 29 (1.1%) | 107 (0.9%) | 344 (0.7%) | 260 (0.7%) | 66 (2.2%) | 120 (2.0%) | 313 (1.7%) | 255 (1.4%) | 40 (2.8%) | 77 (2.0%) | 238 (1.6%) | 170 (1.0%) |
| Antihypertensives | 2271 (83.2%) | 9769 (82.0%) | 38236 (78.4%) | 27713 (72.9%) | 1460 (49.7%) | 2792 (45.8%) | 7285 (40.2%) | 6208 (33.1%) | 604 (42.8%) | 1641 (42.2%) | 5433 (36.1%) | 4737 (27%) |

**Abbreviations:** COPD, chronic obstructive pulmonary disease; HDL-C, high-density lipoprotein cholesterol; HK, Hong Kong; IQR, interquartile range; LDL-C, low-density lipoprotein cholesterol; non-HDL-C, non–high-density lipoprotein cholesterol; NZ, New Zealand; SD, standard deviation; SLE, systemic lupus erythematosus and connective tissue disorders; TC, total cholesterol; UK, United Kingdom.
* Variable available for UK cohort
\# Variable available in HK and NZ cohorts

**Table B:** Number of events, patients, person-years and crude incidence rates in each cohort according to achieved LDL and non-HDL cholesterol category at the year 2 landmark.

|  |  | **Hong Kong** | | | | **New Zealand** | | | | **United Kingdom** | | | |
| --- | --- | --- | --- | --- | --- | --- | --- | --- | --- | --- | --- | --- | --- |
| **Outcome** | | No. of events | No. of patients | No. of person-years | Crude incidence rate, per 1000 p-y | No. of events | No. of patients | No. of person-years | Crude incidence rate, per 1000 p-y | No. of events | No. of patients | No. of person-years | Crude incidence rate, per 1000 p-y |
| **LDL cholesterol, mmol/L** | | | | | | | | | | | | | |
| MACE | | | | | | | | | | | | | |
|  | <1.4 | 204 | 2,731 | 17,101 | 11.93 (10.29 - 13.57) | 86 | 1,413 | 4,662 | 18.45 (14.55 - 22.35) | 144 | 2,940 | 15,236 | 9.45 (8.03 - 11.13) |
|  | 1.4-1.7 | 741 | 11,911 | 74,889 | 9.89 (9.18 - 10.61) | 187 | 3,890 | 13,606 | 13.74 (11.77 - 15.71) | 329 | 6,092 | 32,430 | 10.14 (9.11 - 11.30) |
|  | 1.8-2.5 | 2,990 | 48,768 | 313,598 | 9.53 (9.19 - 9.88) | 580 | 15,060 | 56,733 | 10.22 (9.39 - 11.06) | 959 | 18,113 | 98,309 | 9.76 (9.16 - 10.39) |
|  | ≥2.6 | 2,541 | 38,001 | 253,555 | 10.02 (9.63 - 10.41) | 643 | 17,531 | 70,222 | 9.16 (8.45 - 9.86) | 1,114 | 18,762 | 100,787 | 11.05 (10.42 - 11.72) |
| Cardiovascular death | | | | | | | | | | | | | |
|  | <1.4 | 27 | 2,731 | 17,473 | 1.55 (0.96 - 2.13) | 5 | 1,413 | 4,662 | 1.07 (0.13 - 2.01) | 28 | 2,940 | 15,236 | 1.31 (0.85 - 2.03) |
|  | 1.4-1.7 | 136 | 11,911 | 76,278 | 1.78 (1.48 - 2.08) | 19 | 3,890 | 13,606 | 1.40 (0.77 - 2.02) | 53 | 6,092 | 32,430 | 1.20 (0.88 - 1.65) |
|  | 1.8-2.5 | 422 | 48,768 | 319,376 | 1.32 (1.2 - 1.45) | 37 | 15,060 | 56,733 | 0.65 (0.44 - 0.86) | 78 | 18,113 | 98,309 | 1.08 (0.89 - 1.30) |
|  | ≥2.6 | 349 | 38,001 | 259,022 | 1.35 (1.21 - 1.49) | 33 | 17,531 | 70,222 | 0.47 (0.31 - 0.63) | 120 | 18,762 | 100,787 | 1.13 (0.94 - 1.36) |
| Coronary heart disease | | | | | | | | | | | | | |
|  | <1.4 | 114 | 2,731 | 17,234 | 6.61 (5.4 - 7.83) | 65 | 1,412 | 4,662 | 13.94 (10.55 - 17.33) | 79 | 2,940 | 15,236 | 5.18 (4.16 - 6.46) |
|  | 1.4-1.7 | 423 | 11,911 | 75,350 | 5.61 (5.08 - 6.15) | 129 | 3,896 | 13,606 | 9.48 (7.84 - 11.12) | 164 | 6,092 | 32,430 | 5.06 (4.34 - 5.89) |
|  | 1.8-2.5 | 1,736 | 48,768 | 315,787 | 5.50 (5.24 - 5.76) | 395 | 15,072 | 56,733 | 6.96 (6.28 - 7.65) | 497 | 18,113 | 98,309 | 5.06 (4.63 - 5.52) |
|  | ≥2.6 | 1,520 | 38,001 | 255,431 | 5.95 (5.65 - 6.25) | 467 | 17,539 | 70,222 | 6.65 (6.05 - 7.25) | 675 | 18,762 | 100,787 | 6.70 (6.21 - 7.22) |
| Stroke | | | | | | | | | | | | | |
|  | <1.4 | 86 | 2,731 | 17,331 | 4.96 (3.91 - 6.01) | 21 | 1,413 | 4,662 | 4.5 (2.58 - 6.43) | 45 | 2,940 | 15,236 | 2.95 (2.20 - 3.96) |
|  | 1.4-1.7 | 295 | 11,911 | 75,788 | 3.89 (3.45 - 4.34) | 60 | 3,890 | 13,606 | 4.41 (3.29 - 5.53) | 126 | 6,092 | 32,430 | 3.89 (3.26 - 4.63) |
|  | 1.8-2.5 | 1,255 | 48,768 | 317,022 | 3.96 (3.74 - 4.18) | 189 | 15,060 | 56,733 | 3.33 (2.86 - 3.81) | 356 | 18,113 | 98,309 | 3.62 (3.26 - 4.02) |
|  | ≥2.6 | 1,033 | 38,001 | 256,942 | 4.02 (3.78 - 4.27) | 179 | 17,531 | 70,222 | 2.55 (2.18 - 2.92) | 325 | 18,762 | 100,787 | 3.22 (2.89 - 3.59) |
| All-cause mortality | | | | | | | | | | | | | |
|  | <1.4 | 263 | 2,731 | 17,473 | 15.05 (13.23 - 16.87) | 73 | 1,413 | 4,662 | 15.66 (12.07 - 19.25) | 143 | 2,940 | 15,236 | 9.39 (7.97 - 11.06) |
|  | 1.4-1.7 | 871 | 11,911 | 76,278 | 11.42 (10.66 - 12.18) | 140 | 3,890 | 13,606 | 10.29 (8.59 - 11.99) | 240 | 6,092 | 32,430 | 7.40 (6.52 - 8.40) |
|  | 1.8-2.5 | 2,792 | 48,768 | 319,376 | 8.74 (8.42 - 9.07) | 374 | 15,060 | 56,733 | 6.59 (5.92 - 7.26) | 603 | 18,113 | 98,309 | 6.13 (5.66 - 6.64) |
|  | ≥2.6 | 2,260 | 38,001 | 259,022 | 8.73 (8.37 - 9.08) | 346 | 17,531 | 70,222 | 4.93 (4.41 - 5.45) | 519 | 18,762 | 100,787 | 5.15 (4.72 - 5.61) |
| **non-HDL cholesterol, mmol/L** | | | | | | | | | | | | | |
| MACE | | | | | | | | | | | | | |
|  | <2.2 | 675 | 10,389 | 64,695 | 10.43 (9.65 - 11.22) | 202 | 3,858 | 12,962 | 15.58 (13.43 - 17.73) | 251 | 5,000 | 26,105 | 9.62 (8.50 - 10.88) |
|  | 2.2-2.5 | 1,080 | 18,366 | 116,324 | 9.28 (8.73 - 9.84) | 241 | 5,586 | 20,100 | 11.99 (10.48 - 13.5) | 374 | 6,881 | 36,856 | 10.15 (9.17 - 11.23) |
|  | 2.6-3.3 | 2,482 | 40,774 | 264,663 | 9.38 (9.01 - 9.75) | 500 | 13,849 | 53,287 | 9.38 (8.56 - 10.21) | 800 | 15,645 | 85,106 | 9.40 (8.77 - 10.07) |
|  | ≥3.4 | 2,239 | 31,882 | 213,461 | 10.49 (10.05 - 10.92) | 553 | 14,601 | 58,874 | 9.39 (8.61 - 10.18) | 1,121 | 18,381 | 98,695 | 11.36 (10.71 - 12.04) |
| Cardiovascular death | | | | | | | | | | | | | |
|  | <2.2 | 108 | 10,389 | 65,906 | 1.64 (1.33 - 1.95) | 15 | 3,858 | 12,962 | 1.16 (0.57 - 1.74) | 28 | 5,000 | 26,105 | 1.07 (0.74 - 1.55) |
|  | 2.2-2.5 | 178 | 18,366 | 118,347 | 1.50 (1.28 - 1.73) | 16 | 5,586 | 20,100 | 0.80 (0.41 - 1.19) | 53 | 6,881 | 36,856 | 1.44 (1.10 - 1.88) |
|  | 2.6-3.3 | 344 | 40,774 | 269,575 | 1.28 (1.14 - 1.41) | 34 | 13,849 | 53,287 | 0.64 (0.42 - 0.85) | 78 | 15,645 | 85,106 | 0.92 (0.73 - 1.14) |
|  | ≥3.4 | 304 | 31,882 | 218,322 | 1.39 (1.24 - 1.55) | 29 | 14,601 | 58,874 | 0.49 (0.31 - 0.67) | 120 | 18,381 | 98,695 | 1.22 (1.02 - 1.45) |
| Coronary heart disease | | | | | | | | | | | | | |
|  | <2.2 | 376 | 10,389 | 65,130 | 5.77 (5.19 - 6.36) | 141 | 3,858 | 12,962 | 10.88 (9.08 - 12.67) | 121 | 5,000 | 26,105 | 4.64 (3.88 - 5.54) |
|  | 2.2-2.5 | 611 | 18,366 | 117,092 | 5.22 (4.8 - 5.63) | 173 | 5,586 | 20,100 | 8.61 (7.32 - 9.89) | 186 | 6,881 | 36,856 | 5.05 (4.37 - 5.83) |
|  | 2.6-3.3 | 1,448 | 40,774 | 266,482 | 5.43 (5.15 - 5.71) | 337 | 13,849 | 53,287 | 6.32 (5.65 - 7) | 424 | 15,645 | 85,106 | 4.98 (4.53 - 5.48) |
|  | ≥3.4 | 1,358 | 31,882 | 215,097 | 6.31 (5.98 - 6.65) | 405 | 14,601 | 58,874 | 6.88 (6.21 - 7.55) | 684 | 18,381 | 98,695 | 6.93 (6.43 - 7.47) |
| Stroke | | | | | | | | | | | | | |
|  | <2.2 | 293 | 10,389 | 65,432 | 4.48 (3.97 - 4.99) | 62 | 3,858 | 12,962 | 4.78 (3.59 - 5.97) | 102 | 5,000 | 26,105 | 3.91 (3.22 - 4.74) |
|  | 2.2-2.5 | 451 | 18,366 | 117,531 | 3.84 (3.48 - 4.19) | 72 | 5,586 | 20,100 | 3.58 (2.75 - 4.41) | 135 | 6,881 | 36,856 | 3.66 (3.09 - 4.34) |
|  | 2.6-3.3 | 1,023 | 40,774 | 267,648 | 3.82 (3.59 - 4.06) | 165 | 13,849 | 53,287 | 3.10 (2.62 - 3.57) | 298 | 15,645 | 85,106 | 3.50 (3.13 - 3.92) |
|  | ≥3.4 | 902 | 31,882 | 216,472 | 4.17 (3.89 - 4.44) | 150 | 14,601 | 58,874 | 2.55 (2.14 - 2.96) | 317 | 18,381 | 98,695 | 3.21 (2.88 - 3.59) |
| All-cause mortality | | | | | | | | | | | | | |
|  | <2.2 | 845 | 10,389 | 65,906 | 12.82 (11.96 - 13.69) | 155 | 3,858 | 12,962 | 11.96 (10.08 - 13.84) | 204 | 5,000 | 26,105 | 7.81 (6.81 - 8.96) |
|  | 2.2-2.5 | 1,139 | 18,366 | 118,347 | 9.62 (9.07 - 10.18) | 179 | 5,586 | 20,100 | 8.91 (7.6 - 10.21) | 309 | 6,881 | 36,856 | 8.38 (7.50 - 9.37) |
|  | 2.6-3.3 | 2,279 | 40,774 | 269,575 | 8.45 (8.11 - 8.8) | 303 | 13,849 | 53,287 | 5.69 (5.05 - 6.33) | 493 | 15,645 | 85,106 | 5.79 (5.30 - 6.33) |
|  | ≥3.4 | 1,923 | 31,882 | 218,322 | 8.81 (8.41 - 9.2) | 296 | 14,601 | 58,874 | 5.03 (4.45 - 5.6) | 499 | 18,381 | 98,695 | 5.06 (4.63 - 5.52) |
| **Abbreviations:** LDL-C, low-density lipoprotein; MACE, major adverse cardiovascular events; non-HDL-C, non-high-density lipoprotein; p-y, person year. | | | | | | | | | | | | | |

**Table C:** Subgroup results for major adverse cardiovascular events in main analysis by non-HDL cholesterol category at the year 2 landmark.

|  |  | **Adjusted hazard ratio (95% CI); I² (%)** | | | | | |
| --- | --- | --- | --- | --- | --- | --- | --- |
| **non-HDL cholesterol, mmol/L** | | **<2.2 vs ≥3.4 mmol/L** | **I² (%)** | **2.2-2.5 vs ≥3.4** | **I² (%)** | **2.6-3.3 vs ≥3.4 mmol/L** | **I² (%)** |
| Main analysis | | 0.75 (0.56, 0.99) | 90.4 | 0.74 (0.62, 0.88) | 82.3 | 0.76 (0.72, 0.79) | 26.5 |
| Age (years) | | | | | | | |
|  | ≥70 | 0.78 (0.55, 1.11) | 84.5 | 0.78 (0.63, 0.98) | 73.5 | 0.85 (0.79, 0.92) | 0 |
|  | <70 | 0.72 (0.51, 1.01) | 90.2 | 0.71 (0.64, 0.79) | 45.8 | 0.72 (0.65, 0.80) | 58.6 |
| Sex | | | | | | | |
|  | Women | 0.72 (0.65, 0.81) | 22.6 | 0.79 (0.65, 0.96) | 72.3 | 0.76 (0.71, 0.82) | 0 |
|  | Men | 0.76 (0.53, 1.09) | 91 | 0.70 (0.59, 0.83) | 68.1 | 0.75 (0.68, 0.83) | 54.8 |
| Statin adherence, within 2 years after statin initiation* | | | | | | | |
|  | ≥80% PDC | 0.85 (0.56, 1.31) | 94.3 | 0.80 (0.58, 1.10) | 91.5 | 0.80 (0.74, 0.85) | 15.3 |
|  | <80% PDC | 0.78 (0.57, 1.06) | 0 | 0.67 (0.51, 0.88) | 0 | 0.73 (0.62, 0.86) | 0 |
| Statin persistence, 2 years after statin initiation* | | | | | | | |
|  | Persistent | 0.84 (0.57, 1.22) | 92.5 | 0.79 (0.59, 1.06) | 89.8 | 0.79 (0.74, 0.83) | 0 |
|  | Not persistent | 0.88 (0.55, 1.43) | 62 | 0.72 (0.55, 0.93) | 0 | 0.78 (0.57, 1.07) | 70.4 |
| Ethnic group (HK) | | | | | | | |
|  | Chinese | 0.68 (0.62, 0.74) | -- | 0.67 (0.62, 0.72) | -- | 0.76 (0.72, 0.80) | -- |
|  | Other ethnicity | 0.63 (0.39, 1.04) | -- | 0.66 (0.44, 0.98) | -- | 0.74 (0.54, 1.00) | -- |
| Ethnic group (NZ) | | | | | | | |
|  | Māori | 1.05 (0.47, 2.31) | -- | 0.88 (0.45, 1.69) | -- | 1.07 (0.69, 1.67) | -- |
|  | non-Māori | 1.00 (0.84, 1.19) | -- | 0.89 (0.76, 1.05) | -- | 0.80 (0.71, 0.91) | -- |
| Statin intensity, index date* | | | | | | | |
|  | Low | 0.68 (0.62, 0.75) | 0 | 0.75 (0.56, 1.01) | 70.7 | 0.77 (0.73, 0.82) | 0 |
|  | Moderate or high | 0.85 (0.53, 1.35) | 92 | 0.75 (0.56, 1.01) | 83.2 | 0.76 (0.68, 0.85) | 24.1 |
| Statin intensity, year 2 landmark* | | | | | | | |
|  | Low | 0.70 (0.60, 0.82) | 12.9 | 0.80 (0.55, 1.17) | 77.6 | 0.79 (0.74, 0.85) | 0 |
|  | Moderate or high | 0.88 (0.59, 1.31) | 89.9 | 0.80 (0.63, 1.02) | 77.5 | 0.80 (0.71, 0.89) | 42.7 |
| Diabetes mellitus | | | | | | | |
|  | Yes | 0.59 (0.54, 0.66) | 0 | 0.62 (0.56, 0.67) | 0 | 0.69 (0.64, 0.74) | 0 |
|  | No | 0.84 (0.65, 1.10) | 85.7 | 0.79 (0.65, 0.95) | 78.6 | 0.80 (0.72, 0.88) | 62.8 |
| Baseline total cholesterol, mmol/L | | | | | | | |
|  | ≥5.8 | 0.64 (0.40, 1.04) | 64.3 | 0.85 (0.53, 1.35) | 91.3 | 0.76 (0.68, 0.84) | 38.0 |
|  | <5.8 | 0.81 (0.61, 1.07) | 82.5 | 0.72 (0.65, 0.79) | 0 | 0.81 (0.75, 0.89) | 0 |
| Baseline LDL cholesterol, mmol/L | | | | | | | |
|  | ≥3.8 | 0.84 (0.72, 0.98) | 0 | 0.82 (0.59, 1.15) | 80.8 | 0.74 (0.69, 0.80) | 0 |
|  | <3.8 | 0.78 (0.58, 1.04) | 83.9 | 0.71 (0.63, 0.80) | 25.5 | 0.81 (0.74, 0.87) | 0 |
| Baseline non-HDL cholesterol, mmol/L | | | | | | | |
|  | ≥4.5 | 0.82 (0.69, 0.96) | 0 | 0.83 (0.57, 1.20) | 82.4 | 0.74 (0.69, 0.79) | 0 |
|  | <4.5 | 0.80 (0.64, 1.00) | 71.8 | 0.74 (0.67, 0.81) | 0 | 0.83 (0.76, 0.90) | 0 |
| **Abbreviations:** CI, confidence interval; HR, hazard ratio; HK, Hong Kong; LDL, low-density lipoprotein; non-HDL, non–high-density lipoprotein; NZ, New Zealand; PDC, proportion of days covered; UK, United Kingdom. * Excludes UK cohort | | | | | | | |

**Table D:** Results for sensitivity analysis by achieved LDL cholesterol category using 1-year and 3-month landmarks for all outcomes (Hong Kong and New Zealand data only).

|  |  | **Main analysis (Year 2 Landmark)** | | **Year 1 Landmark** | | **Month 3 Landmark** | |
| --- | --- | --- | --- | --- | --- | --- | --- |
| **Outcome** | **LDL cholesterol, mmol/L** | **Adjusted hazard ratio (95% CI)** | **I² (%)** | **Adjusted hazard ratio (95% CI)** | **I² (%)** | **Adjusted hazard ratio (95% CI)** | **I² (%)** |
| MACE | | | | | | | |
|  | <1.4 | 0.85 (0.58, 1.26) | 90.7 | 0.98 (0.56, 1.72) | 93.9 | 1.06 (0.58, 1.94) | 92.8 |
|  | 1.4-1.7 | 0.80 (0.64, 1.01) | 86.8 | 0.80 (0.61, 1.04) | 92.8 | 0.94 (0.54, 1.63) | 96.1 |
|  | 1.8-2.5 | 0.81 (0.74, 0.88) | 61.4 | 0.84 (0.77, 0.93) | 78.7 | 0.85 (0.69, 1.06) | 86.1 |
|  | ≥2.6 | 1.00 (Reference) | -- | 1.00 (Reference) | -- | 1.00 (Reference) |  |
| Cardiovascular death | | | | | | | |
|  | <1.4 | 0.62 (0.46, 0.84) | 0 | 0.77 (0.59, 1.00) | 0 | 0.95 (0.39, 2.32) | 64.6 |
|  | 1.4-1.7 | 0.87 (0.60, 1.25) | 60.2 | 0.63 (0.53, 0.74) | 0 | 0.57 (0.42, 0.77) | 0 |
|  | 1.8-2.5 | 0.76 (0.67, 0.86) | 0 | 0.75 (0.67, 0.84) | 0 | 0.75 (0.62, 0.90) | 0 |
|  | ≥2.6 | 1.00 (Reference) | -- | 1.00 (Reference) | -- | 1.00 (Reference) | -- |
| Coronary heart disease | | | | | | | |
|  | <1.4 | 0.86 (0.53, 1.40) | 90.7 | 0.82 (0.46, 1.47) | 94.6 | 1.06 (0.64, 1.78) | 85.6 |
|  | 1.4-1.7 | 0.76 (0.57, 1.02) | 86.4 | 0.78 (0.59, 1.02) | 90.1 | 0.93 (0.54, 1.60) | 94.2 |
|  | 1.8-2.5 | 0.77 (0.68, 0.87) | 73.4 | 0.81 (0.73, 0.91) | 73.4 | 0.82 (0.68, 0.97) | 71.7 |
|  | ≥2.6 | 1.00 (Reference) | -- | 1.00 (Reference) | -- | 1.00 (Reference) | -- |
| Stroke | | | | | | | |
|  | <1.4 | 0.77 (0.65, 0.92) | 0 | 0.95 (0.60, 1.52) | 89.1 | 1.01 (0.43, 2.38) | 88.9 |
|  | 1.4-1.7 | 0.86 (0.65, 1.12) | 82.2 | 0.84 (0.65, 1.08) | 79.1 | 0.91 (0.51, 1.61) | 87.9 |
|  | 1.8-2.5 | 0.89 (0.78, 1.02) | 64 | 0.88 (0.77, 0.99) | 65.1 | 0.90 (0.63, 1.27) | 82.9 |
|  | ≥2.6 | 1.00 (Reference) | -- | 1.00 (Reference) | -- | 1.00 (Reference) | -- |
| All-cause mortality | | | | | | | |
|  | <1.4 | 1.21 (0.95, 1.54) | 75.5 | 1.07 (0.89, 1.30) | 68.3 | 0.93 (0.77, 1.12) | 0 |
|  | 1.4-1.7 | 1.00 (0.83, 1.22) | 81 | 0.92 (0.72, 1.17) | 89.7 | 0.79 (0.71, 0.88) | 0 |
|  | 1.8-2.5 | 0.90 (0.79, 1.02) | 81.4 | 0.90 (0.83, 0.98) | 60.2 | 0.86 (0.80, 0.92) | 0 |
|  | ≥2.6 | 1.00 (Reference) | -- | 1.00 (Reference) | -- | 1.00 (Reference) | -- |
| **Abbreviations:** CI, confidence interval; LDL, low-density lipoprotein; MACE, major adverse cardiovascular events. | | | | | | | |

**Table E:** Results for sensitivity analysis for non-HDL cholesterol using year 1 and month 3 landmarks for all outcomes (Hong Kong and New Zealand data only).

|  |  | **Main analysis (Year 2 Landmark)** | | **Year 1 Landmark** | | **Month 3 Landmark** | |
| --- | --- | --- | --- | --- | --- | --- | --- |
| **Outcome** | **non-HDL cholesterol, mmol/L** | **Adjusted hazard ratio (95% CI)** | **I² (%)** | **Adjusted hazard ratio (95% CI)** | **I² (%)** | **Adjusted hazard ratio (95% CI)** | **I² (%)** |
| MACE | | | | | | | |
|  | <2.2 | 0.74 (0.56, 0.99) | 90.4 | 0.82 (0.62, 1.08) | 95.7 | 0.88 (0.50, 1.56) | 96.1 |
|  | 2.2-2.5 | 0.73 (0.62, 0.88) | 83 | 0.82 (0.65, 1.02) | 93.7 | 0.85 (0.55, 1.30) | 94.3 |
|  | 2.6-3.3 | 0.75 (0.72, 0.79) | 34.3 | 0.83 (0.74, 0.93) | 74.9 | 0.82 (0.71, 0.95) | 63.8 |
|  | ≥3.4 | 1.00 (Reference) | -- | 1.00 (Reference) | -- | 1.00 (Reference) | -- |
| Cardiovascular death | | | | | | | |
|  | <2.2 | 0.64 (0.48, 0.84) | 39.9 | 0.76 (0.57, 1.00) | 50.4 | 0.73 (0.37, 1.43) | 67.4 |
|  | 2.2-2.5 | 0.72 (0.62, 0.85) | 0 | 0.65 (0.55, 0.76) | 0 | 0.55 (0.42, 0.73) | 0 |
|  | 2.6-3.3 | 0.69 (0.59, 0.80) | 19.6 | 0.78 (0.69, 0.89) | 0 | 0.75 (0.61, 0.92) | 0 |
|  | ≥3.4 | 1.00 (Reference) | -- | 1.00 (Reference) | -- | 1.00 (Reference) | -- |
| Coronary heart disease | | | | | | | |
|  | <2.2 | 0.70 (0.48, 1.02) | 91.1 | 0.83 (0.60, 1.15) | 97.7 | 0.84 (0.51, 1.39) | 92.7 |
|  | 2.2-2.5 | 0.70 (0.55, 0.90) | 85.9 | 0.82 (0.63, 1.06) | 93.1 | 0.81 (0.60, 1.10) | 83.6 |
|  | 2.6-3.3 | 0.72 (0.65, 0.79) | 51.2 | 0.82 (0.71, 0.95) | 73.2 | 0.78 (0.72, 0.85) | 0 |
|  | ≥3.4 | 1.00 (Reference) | -- | 1.00 (Reference) | -- | 1.00 (Reference) | -- |
| Stroke |  |  |  |  |  |  |  |
|  | <2.2 | 0.80 (0.66, 0.98) | 60.2 | 0.81 (0.52, 1.24) | 82.9 | 0.96 (0.44, 2.08) | 92.9 |
|  | 2.2-2.5 | 0.77 (0.63, 0.93) | 67.5 | 0.83 (0.65, 1.06) | 80.2 | 0.94 (0.46, 1.93) | 93.1 |
|  | 2.6-3.3 | 0.85 (0.73, 0.99) | 68.3 | 0.84 (0.75, 0.95) | 29.1 | 0.92 (0.65, 1.31) | 78.4 |
|  | ≥3.4 | 1.00 (Reference) | -- | 1.00 (Reference) | -- | 1.00 (Reference) | -- |
| All-cause mortality | | | | | | | |
|  | <2.2 | 0.97 (0.83, 1.14) | 70.2 | 0.91 (0.75, 1.09) | 80.8 | 0.79 (0.67, 0.93) | 32.8 |
|  | 2.2-2.5 | 0.95 (0.74, 1.21) | 92.3 | 0.81 (0.76, 0.86) | 0 | 0.80 (0.73, 0.89) | 0 |
|  | 2.6-3.3 | 0.83 (0.74, 0.92) | 65.5 | 0.85 (0.81, 0.89) | 0 | 0.83 (0.77, 0.90) | 0 |
|  | ≥3.4 | 1.00 (Reference) | -- | 1.00 (Reference) | -- | 1.00 (Reference) | -- |
| **Abbreviations:** CI, confidence interval; MACE, major adverse cardiovascular events; non-HDL, non–high-density lipoprotein. | | | | | | | |

**Table F:** Sensitivity analysis of UK cohort with additional adjustments for variables available in UK CPRD and after multiple imputation of 10 datasets. Cox regression models in the main analysis were adjusted for age, sex, hypertension, atrial fibrillation, diabetes mellitus, dyslipidemia, obesity, smoking, alcohol misuse, rheumatoid arthritis, systemic lupus erythematosus and connective tissue disorders, severe mental illness, antihypertensive drugs, antidiabetic drugs, antiplatelet drugs and anticoagulants. Additional variables that were adjusted for in the sensitivity analysis were body mass index, systolic blood pressure, diastolic blood pressure and family history of cardiovascular disease.

|  |  | **Main analysis** | **Multiple imputation and additional covariates** |
| --- | --- | --- | --- |
| **Outcome** | **LDL cholesterol, mmol/L** | **Adjusted hazard ratio (95% CI)** | **Adjusted hazard ratio (95% CI)** |
| MACE |  |  |  |
|  | <1.4 | 0.63 (0.53, 0.75) | 0.63 (0.53, 0.75) |
|  | 1.4-1.7 | 0.72 (0.63, 0.82) | 0.72 (0.63, 0.81) |
|  | 1.8-2.5 | 0.76 (0.70, 0.83) | 0.76 (0.70, 0.83) |
|  | ≥2.6 | 1.00 (Reference) | 1.00 (Reference) |
| Cardiovascular death | |  |  |
|  | <1.4 | 0.59 (0.36, 0.97) | 0.59 (0.36, 0.97) |
|  | 1.4-1.7 | 0.66 (0.45, 0.96) | 0.65 (0.45, 0.95) |
|  | 1.8-2.5 | 0.73 (0.56, 0.96) | 0.74 (0.56, 0.96) |
|  | ≥2.6 | 1.00 (Reference) | 1.00 (Reference) |
| Coronary heart disease | |  |  |
|  | <1.4 | 0.60 (0.47, 0.77) | 0.60 (0.47, 0.76) |
|  | 1.4-1.7 | 0.62 (0.52, 0.74) | 0.62 (0.52, 0.74) |
|  | 1.8-2.5 | 0.68 (0.60, 0.76) | 0.67 (0.60, 0.76) |
|  | ≥2.6 | 1.00 (Reference) | 1.00 (Reference) |
| Stroke |  |  |  |
|  | <1.4 | 0.69 (0.50, 0.96) | 0.69 (0.50, 0.96) |
|  | 1.4-1.7 | 0.94 (0.76, 1.16) | 0.94 (0.76, 1.16) |
|  | 1.8-2.5 | 0.95 (0.81, 1.10) | 0.95 (0.81, 1.10) |
|  | ≥2.6 | 1.00 (Reference) | 1.00 (Reference) |
| All-cause mortality | |  |  |
|  | <1.4 | 1.12 (0.92, 1.37) | 1.12 (0.92, 1.36) |
|  | 1.4-1.7 | 0.99 (0.85, 1.16) | 0.99 (0.85, 1.16) |
|  | 1.8-2.5 | 0.96 (0.86, 1.08) | 0.96 (0.85, 1.08) |
|  | ≥2.6 | 1.00 (Reference) | 1.00 (Reference) |
| **Outcome** | **non-HDL cholesterol** | **Adjusted hazard ratio (95% CI)** | **Adjusted hazard ratio (95% CI)** |
| MACE |  |  |  |
|  | <2.2 | 0.62 (0.54, 0.71) | 0.61 (0.54, 0.71) |
|  | 2.2-2.5 | 0.69 (0.61, 0.78) | 0.69 (0.61, 0.78) |
|  | 2.6-3.3 | 0.72 (0.65, 0.78) | 0.71 (0.65, 0.78) |
|  | ≥3.4 | 1.00 (Reference) | 1.00 (Reference) |
| Cardiovascular death | |  |  |
|  | <2.2 | 0.47 (0.31, 0.72) | 0.47 (0.30, 0.72) |
|  | 2.2-2.5 | 0.72 (0.51, 1.00) | 0.70 (0.50, 0.98) |
|  | 2.6-3.3 | 0.57 (0.43, 0.77) | 0.58 (0.43, 0.77) |
|  | ≥3.4 | 1.00 (Reference) | 1.00 (Reference) |
| Coronary heart disease | |  |  |
|  | <2.2 | 0.54 (0.44, 0.66) | 0.54 (0.44, 0.66) |
|  | 2.2-2.5 | 0.61 (0.51, 0.72) | 0.61 (0.52, 0.72) |
|  | 2.6-3.3 | 0.65 (0.57, 0.73) | 0.65 (0.57, 0.73) |
|  | ≥3.4 | 1.00 (Reference) | 1.00 (Reference) |
| Stroke |  |  |  |
|  | <2.2 | 0.85 (0.68, 1.08) | 0.85 (0.67, 1.07) |
|  | 2.2-2.5 | 0.85 (0.69, 1.04) | 0.84 (0.68, 1.03) |
|  | 2.6-3.3 | 0.92 (0.79, 1.08) | 0.92 (0.78, 1.08) |
|  | ≥3.4 | 1.00 (Reference) | 1.00 (Reference) |
| All-cause mortality | |  |  |
|  | <2.2 | 0.95 (0.80, 1.13) | 0.95 (0.80, 1.13) |
|  | 2.2-2.5 | 1.11 (0.96, 1.28) | 1.11 (0.96, 1.29) |
|  | 2.6-3.3 | 0.91 (0.80, 1.03) | 0.90 (0.80, 1.03) |
|  | ≥3.4 | 1.00 (Reference) | 1.00 (Reference) |
| **Abbreviations:** CI, confidence interval; CPRD, Clinical Practice Research Datalink; LDL, low-density lipoprotein; MACE, major adverse cardiovascular events; non-HDL, non–high-density lipoprotein; UK, United Kingdom. | | | |

**Table G:** Association of low-density lipoprotein cholesterol categories used in the FOURIER study and risk of cardiovascular outcomes and mortality.

| **Outcome** | **LDL cholesterol, mmol/L** | **Adjusted hazard ratio (95% CI)** | **I² (%)** |
| --- | --- | --- | --- |
| MACE |  |  |  |
|  | <0.5 | NE | -- |
|  | 0.5-1.2 | 1.07 (0.68, 1.70) | 87.9 |
|  | 1.3-1.7 | 0.86 (0.60, 1.23) | 93.7 |
|  | 1.8-2.5 | 0.84 (0.75, 0.94) | 70.2 |
|  | ≥2.6 | 1.00 (Reference) | -- |
| Cardiovascular death | |  |  |
|  | <0.5 | NE | -- |
|  | 0.5-1.2 | 0.66 (0.41, 1.05) | 0 |
|  | 1.3-1.7 | 1.00 (0.58, 1.73) | 70.6 |
|  | 1.8-2.5 | 0.77 (0.67, 0.89) | 0 |
|  | ≥2.6 | 1.00 (Reference) | -- |
| Coronary heart disease | |  |  |
|  | <0.5 | NE | -- |
|  | 0.5-1.2 | 1.10 (0.65, 1.88) | 86.6 |
|  | 1.3-1.7 | 0.85 (0.57, 1.28) | 92.7 |
|  | 1.8-2.5 | 0.81 (0.74, 0.88) | 34.8 |
|  | ≥2.6 | 1.00 (Reference) | -- |
| Stroke |  |  |  |
|  | <0.5 | NE | -- |
|  | 0.5-1.2 | 0.90 (0.64, 1.25) | 34 |
|  | 1.3-1.7 | 0.81 (0.56, 1.18) | 81.5 |
|  | 1.8-2.5 | 0.87 (0.72, 1.06) | 68.1 |
|  | ≥2.6 | 1.00 (Reference) | -- |
| All-cause mortality | |  |  |
|  | <0.5 | 0.92 (0.13, 6.74) | 0 |
|  | 0.5-1.2 | 1.36 (0.99, 1.87) | 71.9 |
|  | 1.3-1.7 | 1.04 (0.72, 1.50) | 91.8 |
|  | 1.8-2.5 | 0.87 (0.73, 1.04) | 80 |
|  | ≥2.6 | 1.00 (Reference) | -- |
| **Abbreviations:** CI, confidence interval; LDL, low-density lipoprotein; MACE, major adverse cardiovascular events; NE, not estimable. | | | |

**Table H.** Comparison of hazard ratios and I² (%) in the pre-specified analysis with all three cohorts and the sensitivity analysis excluding the New Zealand cohort.

|  | | **Adjusted hazard ratio (95% CI)** | | **I² (%)** | |
| --- | --- | --- | --- | --- | --- |
| **Outcome** | | **Pre-specified analysis** | **Excluding New Zealand** | **Pre-specified analysis** | **Excluding New Zealand** |
| **LDL cholesterol, mmol/L** | | | | | |
| Major adverse cardiovascular events | | | | | |
|  | <1.4 | 0.82 (0.60, 1.12) | 0.71 (0.57, 0.90) | 90 | 75 |
|  | 1.4-1.7 | 0.79 (0.66, 0.94) | 0.72 (0.67, 0.77) | 87 | 0 |
|  | 1.8-2.5 | 0.80 (0.77, 0.83) | 0.79 (0.75, 0.82) | 0 | 0 |
|  | ≥2.6 | 1.00 (Reference) | 1.00 (Reference) | -- | -- |
| Cardiovascular death | | | | | |
|  | <1.4 | 0.75 (0.47, 1.19) | 0.59 (0.43, 0.81) | 67 | 0 |
|  | 1.4-1.7 | 0.93 (0.59, 1.44) | 0.78 (0.64, 0.95) | 85 | 11 |
|  | 1.8-2.5 | 0.78 (0.69, 0.87) | 0.75 (0.66, 0.86) | 0 | 0 |
|  | ≥2.6 | 1.00 (Reference) | 1.00 (Reference) | -- | -- |
| Coronary heart disease | | | | | |
|  | <1.4 | 0.79 (0.58, 1.08) | 0.69 (0.54, 0.88) | 84 | 59 |
|  | 1.4-1.7 | 0.74 (0.60, 0.92) | 0.68 (0.60, 0.76) | 85 | 25 |
|  | 1.8-2.5 | 0.76 (0.69, 0.83) | 0.73 (0.64, 0.84) | 65 | 77 |
|  | ≥2.6 | 1.00 (Reference) | 1.00 (Reference) | -- | -- |
| Stroke | | | | | |
|  | <1.4 | 0.82 (0.66, 1.03) | 0.75 (0.62, 0.90) | 46 | 0 |
|  | 1.4-1.7 | 0.87 (0.66, 1.15) | 0.79 (0.57, 1.09) | 86 | 85 |
|  | 1.8-2.5 | 0.88 (0.79, 0.97) | 0.86 (0.74, 1.01) | 57 | 70 |
|  | ≥2.6 | 1.00 (Reference) | 1.00 (Reference) | -- | -- |
| All-cause mortality | | | | | |
|  | <1.4 | 1.06 (0.96, 1.16) | 1.07 (0.96, 1.19) | 0 | 0 |
|  | 1.4-1.7 | 1.01 (0.83, 1.22) | 0.91 (0.81, 1.03) | 87 | 52 |
|  | 1.8-2.5 | 0.89 (0.79, 1.00) | 0.87 (0.73, 1.04) | 81 | 86 |
|  | ≥2.6 | 1.00 (Reference) | 1.00 (Reference) | -- | -- |
| **non-HDL cholesterol, mmol/L** | | | | | |
| Major adverse cardiovascular events | | | | | |
|  | <2.2 | 0.73 (0.57, 0.93) | 0.66 (0.61, 0.71) | 92 | 0 |
|  | 2.2-2.5 | 0.72 (0.63, 0.81) | 0.67 (0.63, 0.71) | 78 | 0 |
|  | 2.6-3.3 | 0.75 (0.72, 0.79) | 0.74 (0.71, 0.78) | 0 | 0 |
|  | ≥3.4 | 1.00 (Reference) | 1.00 (Reference) | -- | -- |
| Cardiovascular death | | | | | |
|  | <2.2 | 0.70 (0.46, 1.05) | 0.59 (0.42, 0.82) | 80 | 50 |
|  | 2.2-2.5 | 0.74 (0.64, 0.87) | 0.72 (0.61, 0.85) | 0 | 0 |
|  | 2.6-3.3 | 0.75 (0.55, 1.01) | 0.66 (0.54, 0.81) | 80 | 40 |
|  | ≥3.4 | 1.00 (Reference) | 1.00 (Reference) | -- | -- |
| Coronary heart disease | | | | | |
|  | <2.2 | 0.67 (0.51, 0.87) | 0.60 (0.50, 0.70) | 89 | 53 |
|  | 2.2-2.5 | 0.67 (0.57, 0.79) | 0.62 (0.57, 0.68) | 77 | 0 |
|  | 2.6-3.3 | 0.71 (0.66, 0.77) | 0.70 (0.62, 0.79) | 38 | 66 |
|  | ≥3.4 | 1.00 (Reference) | 1.00 (Reference) | -- | -- |
| Stroke | | | | | |
|  | <2.2 | 0.85 (0.66, 1.08) | 0.75 (0.62, 0.92) | 80 | 56 |
|  | 2.2-2.5 | 0.78 (0.64, 0.93) | 0.74 (0.58, 0.94) | 72 | 76 |
|  | 2.6-3.3 | 0.85 (0.74, 0.98) | 0.83 (0.68, 1.00) | 70 | 77 |
|  | ≥3.4 | 1.00 (Reference) | 1.00 (Reference) | -- | -- |
| All-cause mortality | | | | | |
|  | <2.2 | 0.98 (0.84, 1.14) | 0.89 (0.83, 0.96) | 78 | 0 |
|  | 2.2-2.5 | 0.93 (0.75, 1.17) | 0.91 (0.63, 1.32) | 92 | 95 |
|  | 2.6-3.3 | 0.85 (0.76, 0.95) | 0.83 (0.70, 0.97) | 78 | 81 |
|  | ≥3.4 | 1.00 (Reference) | 1.00 (Reference) | -- | -- |

**Abbreviations:** CI, confidence interval; LDL, low-density lipoprotein; non-HDL, non–high-density lipoprotein.
**Fig A:** Forest plot for major adverse cardiovascular events associated with on-treatment category of low-density lipoprotein (LDL) cholesterol for each cohort. The reference category is LDL cholesterol ≥2.6 mmol/L.
**Abbreviations:** CI, confidence interval; HK, Hong Kong; HR, hazard ratio; NZ, New Zealand; UK, United Kingdom.

**Fig B:** Forest plot for major adverse cardiovascular events associated with on-treatment category of non-high-density lipoprotein (non-HDL) cholesterol for each cohort. The reference category is non-HDL cholesterol ≥3.4 mmol/L.
**Abbreviations:** CI, confidence interval; HK, Hong Kong; HR, hazard ratio; NZ, New Zealand; UK, United Kingdom.

**Fig C:** Forest plot for all-cause mortality associated with on-treatment category of low-density lipoprotein (LDL) cholesterol for each cohort. The reference category is LDL cholesterol ≥2.6 mmol/L.
**Abbreviations:** CI, confidence interval; HK, Hong Kong; HR, hazard ratio; NZ, New Zealand; UK, United Kingdom.

**Fig D:** Forest plot for all-cause mortality associated with on-treatment category of non-high-density lipoprotein (non-HDL) cholesterol for each cohort. The reference category is non-HDL cholesterol ≥3.4 mmol/L.
**Abbreviations:** CI, confidence interval; HK, Hong Kong; HR, hazard ratio; NZ, New Zealand; UK, United Kingdom.
